# Dietary Bioactive Compounds and Their Role in Allergy Prevention: A Comprehensive Review

**DOI:** 10.3390/nu17223506

**Published:** 2025-11-09

**Authors:** Pilar Zafrilla, Pura Ballester, Desirée Victoria-Montesinos, Begoña Cerdá, Javier Marhuenda, Raúl Arcusa, Ana María García-Muñoz

**Affiliations:** Faculty of Pharmacy and Nutrition, UCAM Universidad Católica de Murcia, 30107 Murcia, Spain; mpzafrilla@ucam.edu (P.Z.); pballester@ucam.edu (P.B.); bcerda@ucam.edu (B.C.); jmarhuenda@ucam.edu (J.M.); rarcusa@ucam.edu (R.A.); amgarcia13@ucam.edu (A.M.G.-M.)

**Keywords:** allergic diseases, bioactive compounds, omega-3 fatty acids, vitamin D, curcumin, ginger, quercetin, epigallocatechin gallate

## Abstract

**Background/Objectives:** Allergic diseases are highly prevalent worldwide and represent a significant public health burden. Current therapies mainly alleviate symptoms without addressing underlying immune dysfunction, which has increased interest in nutritional bioactive compounds as preventive or modulatory agents. This review summarizes evidence on omega-3 polyunsaturated fatty acids, vitamin D, curcumin, ginger bioactives, quercetin, and epigallocatechin gallate (EGCG) in allergy prevention and management. **Methods:** A comprehensive literature search was conducted in PubMed, Scopus, and Web of Science up to July 2025, including preclinical and clinical studies reporting immunological, mechanistic, and clinical outcomes. **Results:** Omega-3 fatty acids modulate Th2 responses, promote regulatory T cells, and generate specialized pro-resolving mediators, with modest clinical benefits observed in pregnancy and early life. Vitamin D contributes to immune tolerance and epithelial integrity, although supplementation trials remain heterogeneous. Curcumin inhibits NF-κB/MAPK signaling, enhances barrier function, and improves allergic rhinitis and dermatitis despite limited bioavailability. Ginger constituents ([6]-gingerol, [6]-shogaol) modulate Th1/Th2 balance, mast-cell activity, and oxidative stress, with early clinical evidence in rhinitis and asthma. Quercetin stabilizes mast cells, inhibits Lyn/PLCγ pathways, and improves rhinitis symptoms in small randomized trials using bioavailable formulations. EGCG stabilizes mast cells, attenuates FcεRI signaling, and reduces airway inflammation in preclinical models, though clinical data are scarce. **Conclusions:** Overall, preclinical findings consistently support the immunomodulatory potential of these compounds, while clinical results are promising but heterogeneous. Standardized formulations, long-term trials, and exploration of synergistic effects are required to confirm efficacy and safety, providing future research directions in allergy prevention.

## 1. Introduction

Allergic diseases are among the most prevalent chronic disorders globally and constitute growing public health concern due to their increasing incidence and substantial impact on quality of life and healthcare systems [1]. These conditions include respiratory allergies (such as asthma and allergic rhinitis (AR)), skin diseases (such as atopic dermatitis (AD)), and food allergies, all triggered by hypersensitivity reactions to otherwise harmless environmental or dietary antigens [2,3].

According to the latest estimates from the Global Burden of Disease Study, in 2019, there were approximately 262 million cases of asthma and 171 million cases of AD worldwide [4]. Although age-standardized prevalence rates have slightly declined over the past three decades, the absolute number of cases has continued to rise, particularly in regions with high socio-demographic indices, partly due to population growth and increased awareness [4]. Regarding food allergy, global estimates suggest that it affects approximately 6.4% of children and 5.1% of adults based on self-reported data, although prevalence drops below 1% when confirmed by oral food challenges [5]. In Europe, lifetime self-reported prevalence varies depending on the specific allergen, with cow’s milk allergy reaching 5.7%, egg 2.4%, and peanut 1.5%, among others, showing regional and age-related heterogeneity [6].

Allergic disorders can produce a wide spectrum of clinical manifestations, ranging from mild skin or gastrointestinal symptoms to life-threatening anaphylaxis [7], and are often associated with comorbidities and reduced quality of life [8,9]. Despite advances in treatment, current management strategies primarily rely on allergen avoidance and symptomatic pharmacological therapy [10]. Current pharmacological management of allergic diseases primarily focuses on symptom relief rather than correction of the underlying immune dysfunction. First-line therapies for conditions such as AR include second-generation non-sedating oral antihistamines and intranasal corticosteroids, with current guidelines recommending the latter for moderate-to-severe cases due to their superior efficacy in reducing nasal symptoms [11,12]. Combination therapy with intranasal corticosteroids and antihistamines may offer additional benefit in selected patients, although advantages over monotherapy are generally limited [12,13]. Systemic corticosteroids are reserved for acute or severe exacerbations because of their well-known adverse effects and are not recommended for long-term management [12,14]. Allergen-specific immunotherapy has shown promise in respiratory allergies, but its applicability to food allergies remains limited and is challenged by issues related to safety, standardization, and patient adherence [15].

In this context, there is growing interest in the role of nutritional and dietary interventions as preventive or modulatory tools in allergic diseases. Bioactive compounds naturally found in foods, such as polyphenols, vitamins, fatty acids, and plant-derived molecules, are being extensively investigated for their potential to influence immune responses and reduce allergic sensitization [16,17]. These substances can exert immunoregulatory effects through multiple pathways, including the modulation of Th1/Th2 balance (T Helper Cells Type 1/Type 2) [18,19], promotion of regulatory T-cell (Treg) activity, and inhibition of pro-inflammatory cytokines and mediators such as interleukin (IL)-4, IL-5, and tumor necrosis factor alfa (TNF-α) [19,20].

Moreover, some dietary bioactives have been shown to interact with cellular signaling pathways involved in inflammation and oxidative stress, including the nuclear factor kappa B (NF-κB) and mitogen-activated protein kinase (MAPK) cascades, which are frequently activated in allergic conditions [21]. By targeting these molecular mechanisms, certain compounds may not only attenuate allergic inflammation but also help restore immune homeostasis [22]. The pleiotropic nature of these bioactives, along with their favorable safety profile, supports their potential utility as accessible, cost-effective, and sustainable options within integrative strategies for allergy prevention, particularly when applied during early life, a period considered critical for immune system programming [22].

Given their widespread availability, relatively low cost, and favorable safety profile, these compounds represent a promising avenue for accessible and sustainable prevention strategies, especially in vulnerable populations. This review provides an overview of selected dietary bioactives with potential roles in the prevention of allergic diseases, summarizing their natural sources, mechanisms of action, and evidence from preclinical and clinical studies.

## 2. Materials and Methods

A comprehensive literature search was carried out to identify studies assessing the role of dietary bioactive compounds in the prevention and management of allergic diseases. The databases PubMed, Scopus, and Web of Science were consulted for articles published up to July 2025, without language restrictions. Although no language restrictions were applied, most included studies were available in English or Spanish. Articles published in other languages were screened and, when potentially relevant, translated using a combination of professional translation tools and consultation with fluent colleagues to ensure accuracy in data extraction.

The search strategy combined general terms related to allergic conditions (asthma, allergic rhinitis, atopic dermatitis, food allergy, and allergic inflammation) with specific keywords referring to bioactive compounds (omega-3 fatty acids, vitamin D, curcumin, ginger, quercetin, and epigallocatechin gallate). Medical Subject Headings (MeSH) and free-text terms were used in PubMed, while equivalent search terms were adapted for Scopus and Web of Science.

In Scopus, the query was applied to the Title, Abstract, and Keyword fields (TITLE-ABS-KEY) and limited to the last five years (2020–2025) to ensure recency and relevance. The final search string was as follows: (“asthma” OR “allergic rhinitis” OR “atopic dermatitis” OR “food allergy” OR “allergic inflammation”) AND (“omega-3 fatty acids” OR “vitamin D” OR “curcumin” OR “ginger” OR “quercetin” OR “epigallocatechin gallate” OR “EGCG”) AND (“immune modulation” OR “Th2 response” OR “mast cell activation” OR “IgE” OR “inflammatory cytokines” OR “clinical trial” OR “preclinical study”).

**Figure 1:**
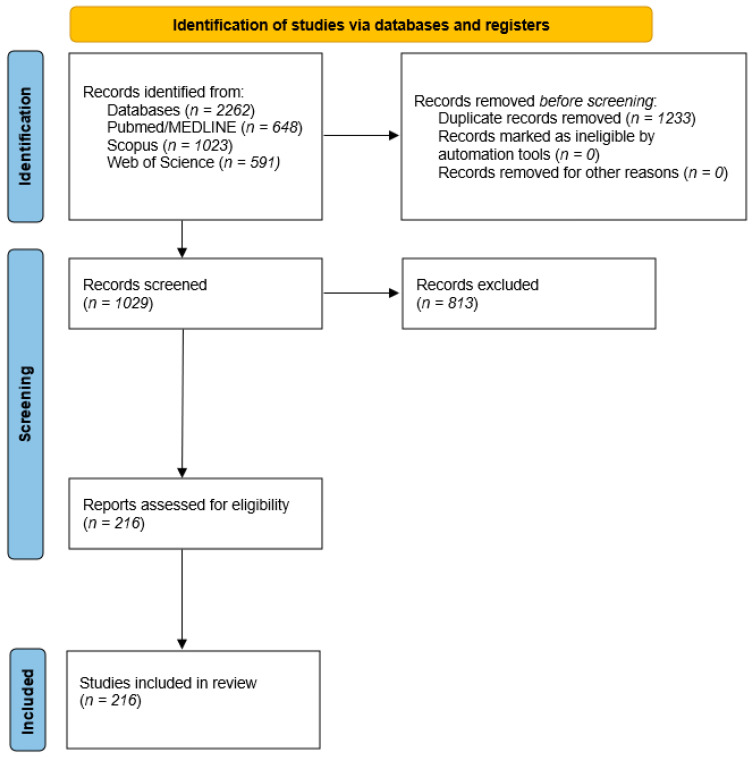
Flow diagram of the literature search and selection process.

Reference lists of eligible papers and relevant reviews were also manually screened to ensure completeness. Although this review was conceived as a narrative synthesis, a PRISMA-style flow diagram (Figure 1) was developed to increase transparency in the identification, screening, and inclusion of studies.

Both preclinical studies, including in vitro and in vivo experiments, and clinical investigations were considered eligible. Recent systematic reviews and meta-analyses were also included when they provided mechanistic insights or clinical outcomes relevant to allergic conditions. In addition, the reference lists of the selected papers and of relevant reviews were screened manually to identify further eligible articles.

Studies were considered for inclusion if they investigated the effects of dietary bioactive compounds on allergic diseases and reported mechanistic, preclinical, or clinical outcomes related to immune modulation, inflammatory markers, oxidative stress, or symptom improvement. Only articles published in peer-reviewed journals were retained for analysis. Exclusion criteria comprised conference abstracts, editorials, letters to the editor, non-peer-reviewed sources, duplicate records, and studies not directly addressing allergy-related outcomes.

The set of bioactive compounds examined in this review (omega-3 polyunsaturated fatty acids, vitamin D, curcumin, ginger bioactives ([6]-gingerol and [6]-shogaol), quercetin, and epigallocatechin gallate (EGCG)) was defined a priori. These compounds were pre-selected on the basis of their documented immunomodulatory or anti-allergic activity (e.g., as detailed in comprehensive reviews of nutraceutical immunomodulators and dietary polyphenols [23]), their relevance as naturally occurring dietary components or common nutritional supplements [17], and the availability of preclinical and clinical data supporting their potential effects on allergic diseases [24].

The overall quality of evidence for each compound was rated qualitatively as High, Moderate, or Low, following a simplified adaptation of the GRADE approach. The classification considered study design (clinical, animal, or in vitro), methodological rigor, reproducibility, relevance to prevention endpoints, and consistency with established immunomodulatory mechanisms.

The evidence collected was synthesized in a narrative manner. For each compound of interest, information was organized into specific sections describing its chemical structure, dietary sources, mechanisms of action, preclinical and clinical evidence, bioavailability, safety, and dosage considerations. This approach allowed for an integrated evaluation of different classes of bioactives, including polyphenols, fatty acids, and vitamins, and their potential contribution to the prevention and management of allergic diseases.

## 3. Bioactive Compounds with Preventive Potential in Allergic Diseases

To provide an overview before addressing each compound individually, the main dietary bioactives considered in this review are summarized in Table 1. The table includes their chemical class and structural features, principal dietary sources, typical intake or supplementation ranges, bioavailability characteristics, and key immunological effects relevant to allergy prevention. This synthesis allows a quick comparison across compounds and facilitates the understanding of their potential roles in modulating allergic diseases.

### 3.1. Omega-3 Polyunsaturated Fatty Acids

#### 3.1.1. Structure and Dietary Sources

Omega-3 fatty acids are a family of polyunsaturated fatty acids (PUFAs) characterized by the presence of the first carbon–carbon double bond between the third and fourth carbon atoms, counting from the methyl (terminal) end of the molecule. This “omega-3” or “n-3” nomenclature is derived precisely from this fundamental structural characteristic [61]. The major omega-3 fatty acids are summarized in Table 2.

The double bonds in omega-3s are in a cis configuration, meaning the adjacent hydrogen atoms are located on the same side of the double bond. This configuration is crucial for the biological properties of these molecules, as it determines their behavior in cell membranes and their susceptibility to oxidation [63].

The double bonds are separated by methylene groups (-CH2-), with exactly two single bonds between each pair of adjacent double bonds. This structural feature is fundamental for enzymatic desaturation and elongation processes.

The main sources are marine fish, microalgae, and specific plant oils, each providing different types of omega-3s. Marine sources are the most direct way to get the long-chain omega-3s, eicosapentaenoic acid (EPA) and docosahexaenoic acid (DHA). These are found in high abundance in oily fishlike salmon, sardines, tuna, mackerel, herring, and anchovies [64]. For individuals who do not consume fish, fish oil and krill oil supplements provide a concentrated source of the omega-3 fatty acids EPA and DHA [65]. Additionally, microalgae and thraustochytrids are a vegan and sustainable source of these same compounds and are increasingly used in the production of commercial supplements [66].

On the other hand, plant sources primarily provide alpha-linolenic acid (ALA). Foods such as flaxseeds, chia seeds, walnuts, and hemp and canola oils are rich in ALA. Although the human body can convert ALA into EPA, this conversion is very inefficient [61]. However, some plants, like those from the Echium and Buglossoides species, produce stearidonic acid (SDA), which the body converts to EPA more efficiently than it converts ALA. Genetically modified plants, such as camelina, are also being developed that can produce EPA and DHA directly [67]. Finally, enriched foods, like certain eggs, dairy, and meats from animals with omega-3-rich diets, can also contribute to the daily intake of these important fatty acids.

#### 3.1.2. Preclinical and Clinical Evidence

##### Preclinical Evidence

Preclinical studies (animal and in vitro models) provide encouraging evidence that omega-3 PUFAs can prevent or reduce allergic diseases by influencing immune regulation and inflammation.

Suppression of allergic responses: omega-3 PUFAs, particularly EPA and DHA, have been shown in animal models to suppress allergic responses, including food allergy and airway inflammation. Their anti-inflammatory effects are mediated by the generation of specialized pro-resolving mediators (SPMs) and Th2 modulation (see Section 3.7 and Table 1) [25,26].

Modulation of immune system: preclinical data indicate omega-3 PUFAs can alter immune cell function, affecting dendritic cells, T cells, B cells, and mast cells, which are central to allergic disease development. They shift immune responses away from Th2 pathways and enhance regulatory mechanisms (see Table 1). [26].

Mechanistic Insights: The capacity of omega-3 PUFAs to act as precursors to SPMs like resolvins and protectins, which actively terminate inflammation, has been demonstrated in animal models of asthma and food allergy, showing reduced airway eosinophilia and improved allergic outcomes (see Section 3.7) [25,68].

Preclinical evidence supports the role of omega-3 PUFAs in preventing allergic diseases by suppressing allergic inflammation and modulating immune responses. However, more research is needed to confirm these effects in humans and to determine effective supplementation strategies.

##### Clinical Evidence

Clinical evidence for omega-3 PUFAs in preventing allergic diseases is mixed, with some benefits in early life but inconsistent results overall. Epidemiological and observational studies suggest that higher maternal fish or omega-3 intake during pregnancy and lactation is associated with a reduced risk of allergic diseases (such as eczema, food allergy, and asthma) in infants and young children. Some randomized controlled trials (RCTs) and meta-analyses show that omega-3 supplementation during pregnancy can reduce the risk of IgE-mediated allergies, eczema, and allergen sensitization in children up to 36 months, but these effects often do not persist beyond early childhood [27]. Higher doses (≥1200 mg/day) and supplementation from pregnancy through lactation may offer greater protection, especially in European populations.

Higher genetically determined levels of omega-3 PUFAs (notably DHA) and a lower omega-6:omega-3 ratio are causally associated with a lower risk of some allergic conditions such as AD and conjunctivitis, but not all allergic diseases. Increasing the omega-6:3 ratio is associated with increased allergic risk [69].

Randomized controlled trials evidence shows small reductions in allergic sensitizations (such as to egg or milk) and atopic conditions, but meta-analyses find the benefits to be modest at best and often not statistically significant for broader outcomes like asthma or AR. The effect size may be influenced by baseline maternal allergy, dietary background, and insufficient dosage/duration [70].

Overall, the clinical evidence over the past decade supports a possible, but not definitive, role for omega-3 PUFA intake (particularly in pregnancy/early childhood) in reducing certain allergic disease risks, especially AD and food sensitization. The benefit is not strong or consistent enough to recommend omega-3 as a universal preventive strategy for all allergic diseases [26,71].

Table 3 summarizes representative preclinical and clinical studies investigating the effects of omega-3 fatty acids on allergic and inflammatory diseases, detailing their main molecular targets, immune responses, and overall direction of effect.

#### 3.1.3. Bioavailability, Safety, and Dosage Considerations

The bioavailability, safety, and dosage of omega-3 fatty acids depend on their chemical form, on the source, and clinical context. The bioavailability of omega-3s (EPA and DHA) varies depending on their chemical form: free fatty acids (FFAs) and phospholipids (PLs, as in krill oil) are more bioavailable than triglycerides (TGs) and ethyl esters (EEs, common in supplements) [72,73]. A meta-analysis, which analyzed 26 high-quality studies, confirmed that triglyceride forms demonstrate superior bioavailability compared to ethyl esters. Fish oils in the form of natural triglycerides result in 50% more plasma EPA and DHA after absorption compared to ethyl esters. This difference occurs because ethyl esters require additional enzymatic processing for absorption, making the process slower and less efficient. Advanced formulations: Emulsions, microencapsulations, and self-emulsifying systems improve oral absorption [74]. Krill oil and fish oil emulsions show better absorption at low doses (<2000 mg) than traditional fish oil [75]. A pharmacokinetic study [76] that evaluated multiple forms of omega-3s confirmed that consumption with dietary fat significantly enhances omega-3 absorption. The studies show that triglyceride absorption increased from 69% to 90% for EPA when taken with high-fat meals. A comprehensive meta-analysis [77] examining all types of omega-3 polyunsaturated fatty acid supplementation reported in randomized trials confirmed that omega-3 supplements are generally well tolerated with predominantly mild side effects. The most frequently reported adverse effects include belching, dyspepsia, nausea, abdominal pain, constipation, and diarrhea, occurring in 1–10% of users according to pharmacovigilance data. Significant safety concerns include the risk of bleeding and atrial fibrillation [78].

The European Food Safety Authority (EFSA) has concluded that supplemental daily intakes of up to 5000 mg of long-chain omega-3 fatty acids do not pose safety concerns for adults. However, a 2022 dose–response analysis published in Prostaglandins, Leukotrienes and Essential Fatty Acids found that doses lower than 3 omega-3 capsules (1 g) per day provided no benefit, except for reduced cardiac mortality only for the equivalent dose of 2 capsules per day [79].

Most health organizations recommend a minimum of 250–500 mg of combined EPA and DHA daily for general health maintenance, according to the National Institutes of Health (NIH) guidelines updated in 2025 [80]. Both EPA and DHA provide health benefits; optimal ratios vary depending on the intended use. A 2021 meta-analysis published in Prostaglandins, Leukotrienes and Essential Fatty Acids found that a balanced 1:1 ratio works well for overall health maintenance [81,82].

Overall, preclinical and clinical findings consistently support the anti-inflammatory and immunomodulatory potential of omega-3 fatty acids, particularly EPA and DHA. However, clinical evidence remains heterogeneous due to variations in dosage, chemical form, and intervention duration. The magnitude of benefit appears modest and context-dependent. Future studies should clarify optimal intake levels, formulation bioavailability, and the long-term preventive impact on allergic disease onset.

### 3.2. Vitamin D

#### 3.2.1. Structure and Dietary Sources

Vitamin D, first described as a vitamin in the early 20th century, is currently considered a prohormone with pleiotropic actions that extend beyond calcium and bone metabolism. A distinctive feature of this compound is its dual origin: it can be synthesized cutaneously upon exposure to ultraviolet B (UVB) radiation or obtained through dietary intake. This duality makes it challenging to establish universal reference intakes, since requirements are strongly influenced by geographic location, season, and lifestyle factors that determine sun exposure [83].

Structurally, vitamin D belongs to the family of fat-soluble secosteroids, characterized by the cleavage of the steroid B-ring that produces a conjugated triene system, conferring photo reactivity. Subsequent hydroxylation reactions generate the active metabolite, 1,25-dihydroxyvitamin D (1,25(OH)_2_D). The two biologically relevant forms are cholecalciferol (vitamin D_3_) and ergocalciferol (vitamin D_2_). While their chemical structures are almost identical, ergocalciferol contains a C22–C23 double bond and an additional methyl group at C24, modifications that slightly alter its metabolic fate and stability compared with cholecalciferol.

Natural dietary sources of vitamin D are limited. Fatty fish such as salmon, mackerel, sardines, and herring, along with fish liver oils, constitute the richest sources of vitamin D_3_. Egg yolks, beef liver, and certain cheeses provide smaller amounts. Analyses indicate that oily fish usually contain between 5–15 µg per 100 g, although species such as Atlantic herring may exceed 40 µg per 100 g. Mushrooms, on the other hand, represent the main plant-based source, as they contain vitamin D_2_; moreover, their content can markedly increase following UV irradiation. Clinical studies have confirmed that consumption of UV-treated mushrooms leads to measurable increases in circulating 25-hydroxyvitamin D, supporting their role as a valuable option in vegetarian or vegan diets [84,85].

Food fortification is widely regarded as the leading public health strategy to improve vitamin D status, particularly in settings where natural dietary sources are scarce. A large body of evidence from systematic reviews and meta-analyses demonstrates that fortifying staple foods such as milk, margarines, breakfast cereals, and plant-based beverages significantly raises circulating 25-hydroxyvitamin D [25(OH)D] levels in both adults and children. The extent of this effect varies according to several factors, including the vehicle used, the form of vitamin D incorporated—D_3_ generally produces greater increases than D_2_—the baseline vitamin D status of the population, and the fortification dose. Overall, pooled analyses indicate that typical fortification programs increase serum 25(OH)D concentrations by approximately 19–25 nmol/L, corresponding to an average rise of around 2 nmol/L for every additional 100 IU (2.5 μg) consumed daily in adults [86,87].

Fortification schemes have also been shown to be cost-effective, sustainable, and acceptable to consumers, with no negative effects on sensory attributes [88]. The choice of food vehicle is critical: dairy products and cereals are among the most effective carriers, although modeling studies suggest that combined strategies—such as the inclusion of both bread and milk—may optimize coverage across diverse dietary patterns [89].

Recent advances emphasize the importance of precision nutrition, recognizing that substantial interindividual variability influences the response to vitamin D fortification. Factors such as age, body mass index, baseline vitamin D status, and genetic polymorphisms can modify absorption and metabolism, ultimately determining the increase achieved. These observations highlight the need for adaptive strategies that consider both individual characteristics and population-specific contexts. To ensure safety and long-term effectiveness, standardized fortification guidelines, regular monitoring, and regionally tailored policies are required [90].

#### 3.2.2. Preclinical and Clinical Evidence

##### Preclinical Evidence

Preclinical investigations strongly suggest that vitamin D exerts immunomodulatory functions that attenuate both the onset and severity of allergic diseases. In preclinical models, vitamin D reinforces epithelial barrier integrity and contributes to immune tolerance mechanisms already summarized in Table 1. Deficiency during gestation or early life has been shown to increase susceptibility to allergic sensitization and to exacerbate disease severity, whereas supplementation mitigates inflammation and restores barrier integrity in models of asthma, AD, and food allergy. These findings underscore the capacity of vitamin D to regulate both innate and adaptive immune responses relevant to allergy pathogenesis, although additional translational studies are required to establish optimal dosage and timing of intervention [28,91,92].

Murine models of AD further highlight vitamin D’s dual role as an immune regulator and barrier stabilizer. In BALB/c and NC/Nga mice sensitized with 2,4-dinitrochlorobenzene or ovalbumin (OVA), administration of calcifediol or topical vitamin D_3_ markedly attenuates dermatitis-like manifestations, including ear swelling, erythema, scaling, and epidermal thickening. These improvements are mechanistically linked to inhibition of the STAT3/AKT/mTOR pathway and reinforcement of cutaneous barrier integrity [93,94].

Vitamin D also modulates inflammatory cascades by suppressing Th2 cytokines (e.g., IL-13, IL-33) and dampening IgE-mediated signaling in dendritic cells, leading to reduced allergic inflammation (see Section 3.7) [29]. Notably, some evidence supports a context-dependent effect, whereby increased local utilization of vitamin D at inflamed skin sites may contribute to the reduced serum concentrations observed in AD, rather than systemic deficiency per se [95,96].

Combination approaches in murine models suggest that vitamin D may act synergistically with topical anti-inflammatory agents, such as crisaborole, to further reduce inflammatory markers, mast cell infiltration, and barrier dysfunction. While animal studies consistently support protective effects, translation to human disease remains incomplete, and large-scale randomized clinical trials are needed to clarify supplementation strategies, appropriate dosing, and patient subgroups most likely to benefit [97,98].

Collectively, preclinical data highlight vitamin D’s capacity to modulate epithelial barrier function, cytokine signaling, and immune tolerance mechanisms involved in the pathogenesis of allergic diseases. The following subsections summarize clinical evidence by disease category.

##### Clinical Evidence

**Respiratory Allergic Diseases**

Vitamin D exerts immunomodulatory effects relevant to the pathogenesis of respiratory allergic diseases such as asthma, AR, and recurrent wheezing. Clinical studies have explored the impact of vitamin D supplementation on respiratory allergic diseases such as asthma, AR, and recurrent wheezing. Additionally, vitamin D enhances epithelial barrier integrity and stimulates antimicrobial peptide synthesis, mechanisms already discussed, which limit allergen penetration and infections that can trigger exacerbations. [28,30,31,32].

Recent clinical evidence indicates that vitamin D supplementation does not reduce asthma exacerbations or improve asthma control in the general pediatric or adult population. However, supplementation may reduce the risk of exacerbations in children with severe vitamin D deficiency (serum 25(OH)D < 10 ng/mL) [33,34].

In the context of AR, vitamin D supplementation has been associated with modest improvements in clinical outcomes, including a reduction in symptom burden and medication requirements, particularly in pediatric populations with documented deficiency. Regarding recurrent wheezing, both prenatal and early postnatal supplementation have been proposed to decrease risk, especially in cohorts characterized by low baseline vitamin D status. However, the observed effect sizes are small, and the overall certainty of the evidence remains limited [99].

Vitamin D supplementation does not significantly alter type 2 inflammatory biomarkers (IgE, eosinophils, fractional exhaled nitric oxide (FeNO), but consistently increases serum IL-10, supporting its anti-inflammatory role. The optimal dosing and timing remain undefined, and routine supplementation is not recommended except in cases of confirmed deficiency or specific high-risk groups [33].

A systematic review and meta-analysis of 13 randomized controlled trials, including more than 1400 children and adults with asthma, evaluated the impact of vitamin D supplementation on inflammatory biomarkers. The results showed that vitamin D supplementation did not significantly affect type 2 inflammatory markers such as serum IgE, blood and sputum eosinophils, or fractional exhaled nitric oxide. However, supplementation was consistently associated with higher serum IL-10 concentrations, an anti-inflammatory cytokine, suggesting a potential immunomodulatory role. Overall, these findings indicate that while vitamin D supplementation does not reduce classical type 2 inflammation in asthma, it may exert anti-inflammatory effects through the upregulation of IL-10. Despite increasing interest in the potential role of vitamin D in respiratory health, current evidence does not provide strong support for its use as a preventive strategy against childhood asthma. The most reliable findings suggest that high-dose maternal supplementation during pregnancy may lower the likelihood of wheezing in children, although a consistent reduction in asthma incidence has not been demonstrated. In contrast, research on supplementation administered directly in infancy or early childhood remains inconclusive, with heterogeneous results and low-certainty evidence. To establish whether vitamin D exerts a meaningful protective effect, additional high-quality randomized controlled trials in pediatric populations are still required [100].

**Type-2 inflammation-predominant skin disorders**

Systematic reviews and meta-analyses consistently show that patients with AD have significantly lower serum 25(OH)D levels than healthy controls, with the deficit most pronounced in children and in severe disease. In pediatric AD, the risk of vitamin D deficiency is approximately doubled, and lower 25(OH)D correlates with higher SCORAD and EASI scores [69].

Randomized controlled trials and meta-analyses further indicate that vitamin D supplementation yields clinically relevant improvements in disease severity across age groups [60]. Pooled analyses report standardized mean differences in Scoring Atopic Dermatitis (SCORAD)/Eczema Area and Severity Index (EASI) ranging from −0.41 to −0.50, with absolute SCORAD reductions often exceeding the minimal clinically important difference [60,69]. Typical regimens provide 1000–1600 IU/day for 1–3 months, though higher doses (e.g., 5000 IU/day) have been studied as adjuvant therapy [70]. Clinical benefit is most evident when serum 25(OH)D reaches ≥20 ng/mL, with no incremental effect observed above this threshold [71].

Improvements are particularly notable in patients with baseline deficiency, in children, and in settings of low ultraviolet exposure. Mechanistically, supplementation upregulates epidermal vitamin D receptor and cathelicidin (LL-37), enhancing barrier function and antimicrobial defense [72] (see Section 3.2.2). Nonetheless, some RCTs have reported no significant differences in severity or type 2 immune biomarkers versus placebo, underscoring the need for large, long-term trials to refine patient selection.

Collectively, current evidence supports vitamin D supplementation as a safe and effective adjunctive therapy for patients with moderate-to-severe AD and documented deficiency [73].

**Food Sensitization and Food Allergy**

Vitamin D modulates immune mechanisms relevant to food sensitization and food allergy during infancy and gestation through several pathways. The active form, 1,25(OH)_2_D, inhibits dendritic cell maturation, thereby reducing the differentiation of naïve T cells into pro-inflammatory effector subsets and promoting the development of regulatory T cells with increased IL-10 production. This shift favors a tolerogenic immune response to dietary antigens [28,92,101], as detailed in Section 3.2.2. Likewise, the enhancement of epithelial barrier integrity and antimicrobial peptide production, as mentioned above, supports mucosal defense and gut microbiota homeostasis [101].

Deficiency in vitamin D during pregnancy and early life impairs these mechanisms, increasing susceptibility to food sensitization and allergy, particularly in the second year of life [102,103]. Genetic polymorphisms affecting vitamin D-binding protein alter vitamin D bioavailability and modify the relationship between serum vitamin D levels and food allergy risk, indicating that individual genetic background influences susceptibility [4]. Collectively, these mechanisms explain how adequate vitamin D status during gestation and infancy supports immune tolerance and barrier function, while deficiency increases the risk of food sensitization and allergy

Despite strong biological plausibility, the latest clinical trial evidence demonstrates that vitamin D supplementation during pregnancy or infancy does not reduce the incidence of food allergy in children. Multiple systematic reviews and meta-analyses of randomized controlled trials, including those with antenatal and early-life supplementation, consistently show no significant effect on the risk of developing food allergy or allergic sensitization in childhood, regardless of dose or timing of intervention [103,104].

Some cohort and case–control studies have reported an association between low maternal or infant vitamin D status and increased risk of food allergy or sensitization, particularly in the second year of life. However, these observational findings are not confirmed by interventional trials. Notably, higher cord blood vitamin D levels have not been shown to confer protection and, in some cases, may even be associated with an increased risk of sensitization. Maternal vitamin D levels during late pregnancy also appear to influence infant allergy outcomes, but findings are inconsistent across studies.

Rueter et al. [105] suggest that, in high-risk infants with sufficient vitamin D levels at birth, early supplementation does not provide additional protection against the development of allergic diseases, highlighting the need to explore alternative preventive strategies.

A meta-analysis [104] of RCTs evaluated the effect of vitamin D supplementation in pregnant women, breastfeeding mothers, and infants for the prevention of allergic diseases. The analysis included several controlled studies and found that prenatal vitamin D supplementation may reduce the risk of childhood wheeze and possibly asthma, especially when higher doses are used during pregnancy, but the effect on asthma is not consistent across all studies and is generally modest. There were no consistent effects observed on AD or allergic sensitization in children. The certainty of the evidence was rated as low to moderate, mainly due to methodological limitations, small sample sizes, and heterogeneity among studies. The authors emphasize that additional large-scale studies with longer follow-up are needed to clarify the potential benefits and risks of vitamin D supplementation for allergy prevention in early life.

Although food allergy prevalence has increased, the translational impact of vitamin D remains uncertain, with conflicting signals from observational studies and neutral RCTs. Beyond its role in bone and calcium metabolism, vitamin D modulates immune responses and may promote tolerance through regulatory T cells and tolerogenic dendritic cells. However, the evidence is inconsistent: some studies link deficiency to a higher risk of food allergy, whereas others suggest that elevated vitamin D levels may increase sensitization. Further randomized controlled trials are needed to clarify its preventive role in childhood food allergy.

In summary, current evidence does not support routine vitamin D supplementation during pregnancy or infancy for the primary prevention of food allergy in children. Further research is needed to clarify whether specific subgroups with severe deficiency or genetic susceptibility might benefit, but no dosing regimen has yet demonstrated efficacy for food allergy prevention in the general population [106].

Table 4 summarizes the main clinical and preclinical evidence regarding vitamin D and its immunomodulatory role in allergic and inflammatory diseases. The table includes human and animal studies describing its molecular mechanisms, target conditions, and overall direction of effect.

#### 3.2.3. Bioavailability, Safety, and Dosage Considerations

The bioavailability of vitamin D is determined not only by the administered dose but also by the formulation, route of delivery, and host-related characteristics. Recent evidence demonstrates that novel delivery systems significantly improve intestinal absorption and serum responses. For example, a randomized controlled trial in healthy adults reported that micellar vitamin D_3_ softgels achieved superior absorption and higher increases in circulating 25(OH)D compared with conventional oil-based formulations, without adverse effects [112]. Likewise, sucrosomial^®^ vitamin D_3_ orodispersible forms produced faster and more sustained rises in serum 25(OH)D than traditional chewable or tablet preparations in vitamin D–deficient individuals [113]. Advanced technologies, such as encapsulation and nanoemulsion systems, have shown very high encapsulation efficiency (>95%) and enhanced systemic bioavailability compared with non-encapsulated vitamin D. In addition, alternative delivery routes are being explored; for instance, transdermal patches containing vitamin D esters may circumvent gastrointestinal absorption challenges and represent a novel therapeutic strategy [114,115].

Beyond formulation, bioavailability is strongly influenced by diet and host factors. Co-ingestion with dietary fat enhances absorption via micelle formation, whereas fat malabsorption syndromes (e.g., celiac disease, inflammatory bowel disease, chronic pancreatitis) reduce intestinal uptake. Obesity is consistently associated with lower circulating 25(OH)D concentrations, likely due to sequestration within adipose tissue. Inter-individual variability is further affected by age, baseline vitamin D status, and genetic polymorphisms in vitamin D–binding proteins. These findings emphasize the relevance of combining improved delivery systems with personalized approaches to optimize vitamin D supplementation [116].

Clinical guidelines currently recommend daily supplementation with 1000–2000 IU (25–50 μg) of cholecalciferol for most healthy adults, which is sufficient to maintain serum 25(OH)D concentrations within the recommended range (≥20 ng/mL and often 30–50 ng/mL) while avoiding toxicity (“Guidelines for Preventing and Treating Vitamin D Deficiency”). Recent narrative reviews suggest that a daily intake of 2000 IU is both safe and effective for preventing and correcting deficiency in the general population, with no significant adverse events in the medium term. Higher intakes of 4000–6000 IU/day have been proposed to sustain serum concentrations of 40–70 ng/mL, although evidence remains inconclusive and further controlled trials are warranted [117,118].

Consensus statements [116] stress that supplementation should be individualized, particularly in groups with increased requirements such as older adults, individuals with obesity, patients with malabsorption, or those with limited sun exposure. Importantly, serum concentrations above 100 ng/mL (≈250 nmol/L) are considered excessive, and values above 150 ng/mL are clearly associated with toxicity, presenting as hypercalcemia, nephrolithiasis, and ectopic calcifications. In clinical practice, the tolerable upper intake level for healthy adults is generally set at 4000 IU/day, although higher doses may be prescribed under supervision for severe deficiency, obesity, or malabsorption (NIH ODS Fact Sheet). Overall, a daily intake of 1000–2000 IU represents a safe and effective strategy for most adults, provided that supplementation is individualized, biochemical monitoring is performed when feasible, and careful surveillance is maintained in patients at risk of hypercalcemia [118,119].

### 3.3. Curcumin

#### 3.3.1. Structure and Dietary Sources

Curcumin is the principal bioactive compound of *Curcuma longa* (turmeric), a rhizomatous plant widely used as a spice and traditional remedy. Chemically, curcumin is a polyphenolic diketone (diferuloylmethane) belonging to the group of curcu-minoids, which also includes demethoxycurcumin and bisdemethoxycurcumin. Its structure consists of two aromatic ring systems containing o-methoxy phenolic groups connected by a seven-carbon linker with α,β-unsaturated carbonyl groups, which confer both antioxidant and anti-inflammatory properties [35].

Turmeric powder contains approximately 2–8% curcuminoids by weight, of which curcumin is the most abundant. Beyond its use as a culinary spice, curcumin is consumed in functional foods, herbal teas, and dietary supplements, often in formulations designed to enhance bioavailability (e.g., complexation with phospholipids, co-administration with piperine, or nanoparticle encapsulation).

In the human diet, turmeric is mainly ingested as a component of curry powders, sauces, and condiments, particularly in South Asian cuisine. However, due to its low natural bioavailability, therapeutic applications of curcumin usually require concentrated extracts or standardized supplements rather than dietary intake alone [35].

#### 3.3.2. Preclinical and Clinical Evidence

##### Preclinical Evidence

Preclinical research provides strong mechanistic support for curcumin as a potential therapy in allergic diseases. Curcumin exerts potent immunomodulatory and antioxidant effects which reduce allergic inflammation by targeting signaling pathways such as NF-κB and MAPK inhibition, and by improving epithelial integrity (see Section 3.7 and Table 1) [35,36,37,40].

Preclinical data also support the application of curcumin in cutaneous allergic disorders. In murine models of AD, oral curcumin supplementation decreased serum IgE concentrations and reduced the severity of dermatitis lesions. These improvements were associated with the suppression of Th2 cytokines (e.g., IL-4 and IL-13) [38,42,120]. Curcumin has also demonstrated ability to inhibit histamine release from mast cells and to downregulate pro-inflammatory cytokine expression in vitro, suggesting mast cell-stabilizing and antihistamine-like activities (see Section 3.7). Evidence from experimental eczema models indicates that curcumin’s antioxidant properties may contribute to the restoration of skin barrier integrity, thus reducing susceptibility to recurrent inflammatory flares.

Collectively, preclinical findings indicate that curcumin not only alleviates clinical manifestations of allergic diseases but also targets underlying immune dysregulation, providing a strong biological rationale for subsequent clinical trials.

##### Clinical Evidence

Although the number of human studies is still limited, clinical research has started to explore the potential role of curcumin as an adjunctive treatment in both respiratory and cutaneous allergic diseases.

**Respiratory Allergy Diseases**

In AR, several clinical trials suggest beneficial effects. Wu and Xiao conducted a randomized, double-blind, placebo-controlled trial including 241 patients with perennial AR who were treated with oral curcumin for two months. The intervention significantly improved nasal symptoms such as sneezing and rhinorrhea and decreased nasal airflow resistance. Immunological analyses showed reductions in IL-4, IL-8, and TNF-α (Tumor Necrosis Factor Alpha), along with increases in IL-10 and soluble ICAM (Intercellular Adhesion Molecule), while no changes were observed in prostaglandin E_2_ or leukotriene C_4_ [36]. More recently, Parameswara Panicker et al. tested a combined formulation of curcumin and ashwagandha (CQAB) in patients with mild AR. After 28 days, CQAB significantly improved the Total Nasal Symptom Score (TNSS), sleep quality, and mood compared with both placebo and a high-bioavailability curcumin formulation (CGM), highlighting a potential synergistic effect of this herbal combination [37]. Additional case evidence also supports curcumin’s potential. Waghray et al. reported a patient with perennial AR and episodic asthma who added bioavailable turmeric to his usual treatment. After two months, the patient reported reduced need for oral steroids and improved symptom control, suggesting an adjunctive benefit [121].

In asthma, evidence from human studies remains scarce. Quan et al. designed a phase 2 randomized, placebo-controlled pilot trial to evaluate curcumin supplementation (1500 mg twice daily for 12 weeks) in patients with moderate to severe asthma. The study aims to assess outcomes including asthma control, forced expiratory volume in 1 s (FEV_1_), blood eosinophils, total IgE, and FeNO [39]. This type of preliminary evidence mirrors the situation described for omega-3, where clinical benefits are promising but not yet consistent or robust.

**Type-2 inflammation-predominant skin disorders**

AD has received particular attention in relation to curcumin. In a systematic review of clinical evidence, Vaughn et al. identified multiple studies in which turmeric extracts, applied topically or taken orally, improved skin condition in AD patients [122]. For example, turmeric-containing creams have been shown to significantly reduce erythema, scaling, and pruritus compared with placebo, with a favourable safety profile and minimal local irritation. These findings are consistent with animal studies and reinforce the therapeutic promise of curcumin in AD.

In urticaria, evidence remains preliminary but promising. De et al. [123] conducted an open-label pilot study in patients with refractory chronic spontaneous urticaria and found that oral curcumin supplementation led to reductions in both wheal frequency and pruritus intensity after eight weeks of treatment. Although limited by its small sample size and absence of placebo control, this study supports the hypothesis that curcumin may act as a mast cell stabilizer with antihistamine-like properties. These results align with in vitro observations of curcumin’s ability to downregulate histamine release and pro-inflammatory cytokines.

In eczema and other inflammatory dermatoses, curcumin has often been tested as part of polyherbal topical formulations. Rawal et al. [124] investigated a polyherbal cream containing curcumin in patients with eczema and found significant reductions in erythema, scaling, and pruritus after four weeks of application. While the design did not allow attribution of effects exclusively to curcumin, these results support the potential role of turmeric-derived compounds in topical dermatological products. Moreover, curcumin’s antioxidant effects may contribute to restoring skin barrier integrity and reducing susceptibility to recurrent flares.

Taken together, clinical evidence indicates that curcumin may improve AR symptoms and modulate immune responses, particularly when delivered in enhanced formulations designed to overcome bioavailability issues. However, as also noted in omega-3 trials, the main limitations include small sample sizes, heterogeneity of formulations, and lack of long-term follow-up.

Preclinical studies robustly demonstrate curcumin’s anti-allergic and anti-inflammatory effects through NF-κB and MAPK inhibition, but translation to clinical efficacy is still limited. Trials are often small, heterogeneous in formulation, and short in duration. While symptom improvements have been reported in AR and dermatitis, dose–response relationships and long-term safety remain unclear. Further standardized, placebo-controlled studies are warranted.

Table 5 summarizes the main clinical and preclinical evidence on curcumin and its derivatives in allergic and inflammatory conditions.

#### 3.3.3. Bioavailability, Safety, and Dosage Considerations

As discussed for omega-3, low oral bioavailability remains the primary translational barrier. Although turmeric and curcumin are widely investigated for anti-inflammatory benefits, multiple reports document allergic contact dermatitis (ACD), contact urticaria, pigmented contact dermatitis, and, more rarely, severe cutaneous adverse reactions. Contemporary dermatology case series and reviews highlight patch-test positivity to turmeric among patients with suspected ACD, including cultural and occupational exposures (e.g., spice milling, use in cosmetics or religious powders such as *kumkum*) [125].

Cultural practices involving direct skin application (e.g., smearing turmeric on jewelry or skin before bathing) have precipitated localized ACD and pigmentary changes [126]. Beyond cutaneous-localized disease, severe eruptions such as acute generalized exanthematous pustulosis have been attributed to oral curcumin, underscoring that systemic exposure can, in rare cases, provoke serious hypersensitivity [127]. Parenteral exposure presents an additional risk: the U.S. Food and Drug Administration (FDA) documented a serious hypersensitivity reaction following intravenous curcumin emulsion, reinforcing that non-oral routes may carry distinct safety concerns [128].

Broader clinical appraisals of topical curcumin note that while many trials target inflammatory dermatoses, adverse events consistent with ACD or contact urticaria are intermittently reported, especially with essential-oil–containing or multi-ingredient formulations [129,130].

The literature therefore presents a double-edged profile: curcumin combines immunomodulatory and antioxidant actions with a potential to act as a contact allergen in sensitive individuals. This duality argues for measured use: topical or oral turmeric products can be considered as adjuncts in inflammatory skin disease, but new or worsening dermatitis (especially at application sites) should prompt patch testing and discontinuation of the suspected product [129,130].

### 3.4. Ginger

#### 3.4.1. Chemical Structure and Dietary Sources

Ginger (*Zingiber officinale*) is rich in potent phenolic bioactive compounds, primarily the phenolic constituents [6]-gingerol and [6]-shogaol, which form the chemical backbone of its antiallergic action [131]. The primary pungent compound in fresh ginger is [6]-gingerol, most abundant in fresh rhizomes. It is a phenolic alkanone featuring a decanone chain attached to a methoxyphenol ring [132]. Upon dehydration, [6]-gingerol turns into [6]-shogaol, which doubles its potency. Additional homologues, including [8]- and [10]-gingerol, and derivatives like zingerone and paradols contribute to their diverse biological activities [133]. Essential oils (e.g., zingiberene, α-curcumene) contribute minor yet modulatory effects. Dietary sources include fresh rhizome (ginger), dried or powdered preparations, standardized extracts, beverages, and supplements [134]. Commercial supplement doses typically range from a few hundred milligrams to several grams of powdered extract. In clinical trials, typical oral dosing ranges from 500 mg to 2 g of standardized extract daily (containing ≥5% gingerols) [135].

#### 3.4.2. Preclinical and Clinical Evidence

##### Preclinical Studies

A comprehensive range of preclinical studies has established the immunomodulatory, anti-inflammatory, and antioxidant properties of ginger (*Zingiber officinale*) and its active metabolites, particularly [6]-gingerol and [6]-shogaol [136,137,138]. In models of AR, dietary administration of ginger significantly decreased the expression of histone deacetylase (HDAC)2 and HDAC3 in Caco-2 cells [43]. The present study suggests the possibility that food ingredients such as ginger modulate vitamin A metabolism in the gut through the regulation of Retinoic Acid (RA) synthesis, which may contribute to RA-mediated regulation of immune responses and the regulation of allergic inflammation [43]. In parallel, splenic cytokines such as IL-4, IL-13, and IFN-γ (Interferon-Gamma) were downregulated, highlighting the capacity of ginger to modulate systemic immune responses [43]. As with curcumin (3.3), [6]-gingerol suppressed T-cell cytokines and inhibited NF-κB signaling in vitro [44].

In murine asthma models, ginger extracts alleviate airway inflammation and reduce eosinophil and neutrophil infiltration. Specifically, [6]-shogaol decreased Th2 cytokine expression, suppressed NF-κB activation, and promoted Treg differentiation through (Cyclic Adenosine Monophosphate (cAMP) signaling. These effects are also linked to phosphodiesterase inhibition and the attenuation of oxidative stress by enhancing antioxidant enzyme expression (see Section 3.7 and Table 1) [139,140].

Other animal models broaden the picture of ginger’s protective role. For example, Shen et al. demonstrated that gingerol-enriched ginger ameliorated neuropathic pain and reduced neuroimmune activation via gut microbiota modulation, mitochondrial regulation, and decreased inflammatory gene expression in colon and spinal cord [141,142]. These findings support the idea of a gut–immune–brain axis, relevant for allergic and inflammatory diseases. Similarly, Li et al. showed that ginger-processed Magnoliae Officinalis Cortex mitigated OVA-induced asthma by modulating gut and lung microbiota and increasing short-chain fatty acids, thereby reducing airway inflammation through the gut–lung axis.

In the context of AD, Neubauer et al. reported potent in vitro inhibition of NF-κB activation and cytokine release by ginger extract in human skin cell cultures. Combined with cannabidiol, ginger extract alleviated dermatitis symptoms in vivo, highlighting potential for topical applications in allergic skin disease [143]. Additional mechanistic evidence shows that ginger enhances expression of ldehyde dehydrogenase 1 family member A1 in intestinal epithelial cells, which promotes RA signaling and may contribute to immune tolerance and regulation of allergic inflammation [43].

Beyond allergy-specific models, reviews of anti-inflammatory herbs emphasize ginger’s broad capacity to suppress inflammatory pathways, including cyclooxygenase (COX), lipoxygenase (LOX), and NF-κB, and to decrease production of TNF-α, IL-1β, and other proinflammatory mediators [144,145]. Collectively, the preclinical evidence demonstrates that ginger acts at multiple levels—mast cell stabilization, Th2 suppression, Treg induction, oxidative stress reduction, and microbiota modulation—supporting its development as a multi-target therapeutic in allergic disease.

##### Clinical Studies

Clinical research, although still at an early stage compared to extensive preclinical literature, increasingly validates the therapeutic potential of ginger in allergic diseases. The best-documented evidence comes from randomized controlled trials in AR. A Thai RCT involving 80 patients compared ginger extract (500 mg/day, standardized to 3% gingerols) with loratadine (10 mg/day) over six weeks. Both interventions significantly improved TNSS, and rhinoconjunctivitis quality of life questionnaire (RQLQ) scores; however, only the ginger group demonstrated significant improvements in nasal airflow measured by acoustic rhinometry. Moreover, ginger was associated with fewer adverse events, particularly reduced rates of sedation, dizziness, and constipation, compared to loratadine [146].

Emerging evidence suggests ginger may also benefit asthma patients. Emala et al. conducted a double-blind RCT in 32 patients with mild-to-moderate asthma, administering 1 g ginger twice daily for 56 days. Ginger improved multiple symptom-related endpoints, including Asthma Control Test (ACT) scores and asthma-specific quality of life, while modulating IL-13 and IL-17A levels [147]. Complementary pharmacokinetic studies in these patients revealed rapid absorption and metabolism of gingerols and shogaols, with enhanced systemic exposure following chronic dosing [148]. These results provide mechanistic support for clinical efficacy while highlighting the importance of considering metabolite activity.

Topical formulations have been tested in allergic dermatitis. Neubauer et al. reported that an oil-in-water emulsion containing ginger extract and cannabidiol significantly reduced pruritus (55% reduction) and improved skin barrier function in 44 patients with AD, with no adverse events [143]. This trial underscores ginger’s potential role in skin-related allergic conditions. Reviews further emphasize that ginger may exert synergistic effects when combined with other natural products, such as cannabidiol or magnolia extracts, amplifying its clinical relevance [149].

Broader systematic reviews and meta-analyses corroborate these findings. Khaki Vaighan et al. summarized multiple lines of evidence and concluded that ginger’s ameliorative effects extend across AR, asthma, and AD, mediated by antioxidant and immunomodulatory mechanisms [150]. Abdelmawgood et al. highlighted mast-cell suppression as a consistent feature across asthma studies, reinforcing ginger’s role in attenuating histamine-driven responses [44]. Meanwhile, meta-analyses in related conditions such as osteoarthritis and obesity demonstrate ginger’s capacity to significantly reduce c-reactive protein (CRP) and TNF-α, indirectly supporting its anti-inflammatory potential in allergy [151].

Collectively, these trials and reviews establish ginger as a promising adjunctive therapy for allergic conditions. While AR has the strongest evidence base, asthma and AD data are growing, with early-phase trials suggesting clinically meaningful benefits.

Table 6 compiles the principal clinical, preclinical, and review-based evidence on the biological actions of ginger-derived compounds, particularly 6-gingerol and 6-shogaol, in allergic and inflammatory conditions.

#### 3.4.3. Bioavailability, Safety, and Dosage Considerations

##### Pharmacokinetics and Formulations

Pharmacokinetic investigations in humans have provided detailed insights into the absorption, distribution, and metabolism of gingerols and shogaols. Zhang et al. administered 1 g ginger root extract twice daily to asthma patients for 56 days and showed that peak plasma concentrations of [6]-gingerol and [6]-shogaol occurred within two hours, with half-lives between 0.6 and 2.4 h. Ketone reduction yielded gingerdiols as major metabolites, while cysteine conjugation accounted for the majority of [6]-shogaol metabolism [148]. Importantly, chronic supplementation increased the absorption and bioavailability of these compounds, suggesting adaptation of metabolic pathways. Gingerols undergo extensive conjugation (glucuronidation, sulfation) leading to low plasma bioavailability. [6]-Shogaol, being more lipophilic, persists longer. Innovative delivery systems—cyclodextrin complexes, liposomes, niosomes, and self-emulsifying nanoparticles—have shown improved stability and sustained release, with preclinical models demonstrating enhanced therapeutic effects.

These findings underscore the importance of formulation strategies. Conventional oral administration may be limited by first-pass metabolism and poor solubility. Innovative approaches—including cyclodextrin inclusion complexes, lipid-based nanoparticles, self-emulsifying drug delivery systems, and liposomes—have shown promise in enhancing bioavailability and sustained release in preclinical studies. Such systems may enable more consistent therapeutic outcomes in clinical settings.

##### Safety and Drug Interactions

Oral intake up to 2 g/day is well tolerated; doses of 500 mg/day for AR were safe over 6 weeks in clinical studies. High doses in animals showed no acute toxicity (no-observed-adverse-effect level, NOAEL > 5 g/kg). Caution is advised with concurrent anticoagulants due to mild antiplatelet effects, and during pregnancy due to limited safety data. Mild gastrointestinal upset may occur at higher doses.

Across preclinical toxicology and clinical trials, ginger has demonstrated an excellent safety profile. In humans, oral intake of up to 2 g/day is well tolerated, with minimal gastrointestinal disturbances as the most common side effect. In AR, 500 mg/day was safely used for six weeks, with a side-effect profile superior to loratadine [146]. Animal studies have shown no acute toxicity at doses greater than 5 g/kg, establishing a wide margin of safety [144].

Caution is warranted with concurrent anticoagulant use, given ginger’s mild antiplatelet activity. In pregnancy, ginger is widely used to treat nausea and vomiting and has been generally recognized as safe, but robust data for long-term use in allergic disease remain limited [152]. Reviews of herbal medicines consistently emphasize ginger’s favorable tolerability relative to conventional anti-inflammatory drugs, which are often associated with systemic side effects [145].

### 3.5. Quercetin

#### 3.5.1. Chemical Structure and Dietary Sources

Quercetin is a naturally occurring flavonol, a subclass of flavonoids, characterized by a C6–C3–C6 skeleton that includes two aromatic rings (A and B) and a central heterocyclic ring (C). This arrangement corresponds to the 3-hydroxyflavone backbone, common to all flavonols. Its molecular formula is C_15_H_10_O_7_, and its IUPAC name is 3,3′,4′,5,7-pentahydroxy-2-phenylchromen-4-one, which highlights the five hydroxyl groups at positions 3, 5, 7, 3′, and 4′ [153].

These hydroxyl groups are responsible for many of quercetin’s biological properties, particularly its antioxidant potential, as they participate in hydrogen donation and metal ion chelation. The catechol group on the B-ring (at positions 3′ and 4′) plays a crucial role in scavenging reactive oxygen species, while the conjugated double bond between C2 and C3, in combination with the carbonyl at C4, contributes to the stabilization of electron delocalization, enhancing its redox activity [154].

In plants, quercetin is typically found in the form of glycosides, where a sugar molecule, such as glucose or rutinose, is attached, usually at the 3-position. The most common natural form is quercetin-3-O-glucoside, whereas the aglycone (non-glycosylated) form is less water-soluble but more lipophilic [155]. Glycosylation improves water solubility and influences absorption and bioavailability in the human body.

Regarding dietary sources, among vegetables, onions (*Allium cepa*) are considered the richest and most extensively studied. The distribution of quercetin within the onion is not uniform, with significantly higher concentrations in the outer layers and skins compared to the inner flesh. Red and yellow varieties generally exhibit the highest levels, with quercetin content reaching up to 486 mg/kg fresh weight [156], due to their role in protecting against ultraviolet radiation and microbial stress. The main glycosides present in onions, quercetin-4′-O-glucoside and quercetin-3,4′-O-diglucoside, are also noted for their favorable bioavailability. Several factors influence the quercetin content in onions, including cultivar type, soil composition, sunlight exposure, post-harvest storage, and cooking methods, with raw onions retaining higher levels than cooked ones [153].

Other vegetables also contribute to dietary intake of quercetin. Leafy greens such as kale (*Brassica oleracea* var. acephala) contain between 20 and 60 mg/kg, spinach (*Spinacia oleracea*) ranges from 15 to 30 mg/kg, and red leaf lettuce from 10 to 25 mg/kg. Cruciferous vegetables like broccoli and Brussels sprouts provide 10–25 mg/kg and 8–20 mg/kg, respectively, with higher concentrations in the florets. Additional contributors include green beans (5–15 mg/kg), cherry tomatoes (3–10 mg/kg), and asparagus (5–12 mg/kg) [153].

Fruits also represent significant sources, especially apples (Malus domestica), which accumulate most of their quercetin in the peel—up to five times more than in the flesh. Quercetin glycosides such as quercetin-3-galactoside are predominant, with values ranging from 182 to 1285 mg/kg in the peel and approximately 6.7 mg/kg in the pulp [157]. Other rich fruit sources include berries. Among them, Saskatoon berries (*Amelanchier alnifolia*) show notably high levels, with quercetin-3-galactoside concentrations between 236 and 307 mg/kg fresh weight [158]. Other berries such as blackcurrants (25–50 mg/kg), wild blueberries (30–50 mg/kg), cranberries (20–40 mg/kg), elderberries (15–35 mg/kg), and blackberries (10–25 mg/kg) also contribute considerably to quercetin intake [153,159].

Herbs and spices, though consumed in smaller amounts, can provide high concentrations of quercetin. Dill (*Anethum graveolens*) contains 50–100 mg/kg, coriander (*Coriandrum sativum*) 40–80 mg/kg, fennel (*Foeniculum vulgare*) 30–70 mg/kg, and lovage (*Levisticum officinale*) 25–60 mg/kg [153]. Beverages like green tea (10–25 mg/L), black tea (5–15 mg/L), red wine (2–10 mg/L), and apple juice (1–5 mg/L, especially if unfiltered) also serve as minor but relevant contributors [156].

Secondary sources include nuts such as almonds (2–5 mg/kg) and walnuts (1–3 mg/kg), grains like buckwheat (5–15 mg/kg) and whole wheat (1–3 mg/kg), and legumes including green lentils (2–5 mg/kg) and black beans (1–4 mg/kg) [159]. The final quercetin content in foods depends on various factors, including agricultural practices (e.g., organic farming, sunlight exposure, fertilization), post-harvest conditions (e.g., storage and processing), and cooking methods (e.g., boiling vs. steaming). Additionally, genetic differences among cultivars and tissue-specific localization (e.g., peel vs. pulp) significantly affect quercetin accumulation [45].

#### 3.5.2. Preclinical and Clinical Evidence

##### Preclinical Evidence

The pharmacological potential of quercetin in allergic conditions has been extensively demonstrated in preclinical studies, and preliminary human data provide further support for its therapeutic utility. In this section, we summarize the current scientific evidence from in vivo animal models and clinical trials, highlighting the immunological and histopathological outcomes observed following quercetin administration.

A wide range of animal models has consistently demonstrated the efficacy of quercetin in attenuating allergic inflammation and clinical symptoms. These models have been instrumental not only in confirming the pharmacodynamic properties of quercetin but also in elucidating its mechanism of action at the tissue, cellular, and molecular levels.

In murine models of AR, quercetin has shown a robust capacity to alleviate nasal symptoms and modulate local immune responses. In one study, BALB/c mice sensitized and challenged with OVA exhibited hallmark features of AR, including frequent sneezing, nasal rubbing, and elevated expression of proangiogenic and proinflammatory cytokines in the nasal mucosa. Oral administration of quercetin at a dose of 25 mg/kg/day for five days resulted in a significant reduction in these symptoms and suppressed the levels of vascular endothelial growth factor, basic fibroblast growth factor, TNF-α, IL-6, and IL-8 in nasal lavage fluid. These findings indicate that quercetin not only modulates acute-phase responses but also interferes with the angiogenic remodeling characteristic of chronic AR [46]. The suppression of angiogenic factors may contribute to long-term control of tissue hyperplasia and inflammatory cell recruitment.

Additional data from a rat model of AR further support these outcomes. In this study, quercetin (80 mg/kg/day) was administered intraperitoneally for seven consecutive days after sensitization and challenge with ovalbumin. The treatment group showed significantly reduced total serum IgE and OVA-specific IgE levels, along with improved histopathological findings in the nasal mucosa, including decreased epithelial disruption, goblet cell hyperplasia, and inflammatory infiltration. Immunohistochemical staining revealed reduced expression of cyclooxygenase-2 and vasoactive intestinal peptide, two mediators involved in vasodilation and epithelial permeability during allergic responses [47].

In models of allergic conjunctivitis, quercetin suppressed mast cell degranulation, eosinophil infiltration, and reduced local levels of inflammatory mediators (histamine, IL-4, TNF-α). These key anti-allergic effects are mechanistically associated with the blockade of the Lyn kinase signaling cascade and downstream targets Lyn tyrosine kinase/phospholipase C gamma/extracellular signal-regulated kinase 1 and 2 (PLCγ/ERK1/2/NF-κB), as confirmed in vitro (see Section 3.7 and Table 1) [160].

Further supporting these findings, Joskova et al. evaluated the acute bronchodilator effect of a single oral dose of quercetin (20 mg/kg) in a guinea pig model of ovalbumin-induced allergic asthma [48]. The authors observed significant improvements in airway conductance, reduced tracheal smooth muscle reactivity, and a marked suppression of histamine- and acetylcholine-induced bronchoconstriction, both in vivo and in vitro. These results underscore the potential of quercetin as a rapid-acting bronchodilator capable of attenuating airway hyperresponsiveness—a cardinal feature of allergic asthma.

In a complementary model of chronic urticaria, Zhao et al. investigated the immunomodulatory properties of quercetin in mice sensitized with ovalbumin via an IgE-mediated pathway [49]. Their findings revealed that quercetin significantly inhibited mast cell degranulation and eosinophil infiltration in dermal tissues, accompanied by decreased serum levels of histamine, IgE, IL-4, and IL-13. Mechanistically, these effects were associated with the upregulation of the inhibitory receptor CD300f and suppression of the PI3K-AKT-NF-κB signaling cascade (see Section 3.7).

Importantly, the limited oral bioavailability of quercetin has spurred interest in enhanced delivery systems. A study investigating various glycosylated derivatives found that enzymatically modified isoquercitrin (EMIQ), which contains an α-oligoglucosyl chain, significantly outperformed native quercetin in the murine passive cutaneous anaphylaxis (PCA) model. Mice receiving EMIQ showed a dose-dependent inhibition of the PCA reaction, and plasma analysis confirmed higher systemic concentrations of quercetin metabolites such as quercetin-3-O-glucuronide and isoquercitrin itself. Notably, while native quercetin and isoquercitrin showed limited efficacy, EMIQ was able to exert significant anti-allergic effects without the need for solubilizing agents [50,51].

Another innovative approach involves quercetin-loaded chitosan nanoparticles (QCS), designed for nasal administration. This formulation demonstrated high encapsulation efficiency (≈80%) and sustained release kinetics. In OVA-induced AR mice, QCS significantly reduced symptom scores, serum IgE, and the expression of IL-6, IL-17, and TNF-α, while promoting histological restoration of nasal epithelium. The strong mucosal adhesion and prolonged residence time of the nanoparticles likely enhanced quercetin’s local bioavailability and efficacy [51].

Finally, systems biology approaches have confirmed quercetin as a key therapeutic compound in herbal formulations traditionally used for allergy treatment. In a network pharmacology study of the Huangqi-Gancao herb pair, quercetin emerged as the primary bioactive agent targeting 57 allergy-related proteins. Experimental validation in OVA-sensitized mice revealed that quercetin, either alone or as part of the herbal decoction, significantly reduced clinical symptoms, total IgE, and restored expression of critical immunoregulatory genes (RELA, IFNG, IRF1, and NFKBIA) within the NF-κB signaling axis [52].

##### Clinical Evidence

Despite robust preclinical findings, clinical evidence supporting the efficacy of quercetin in allergic conditions remains limited. Nonetheless, two recent randomized controlled trials have provided promising data, particularly in the context of persistent AR.

In the first study, conducted by Di Pierro et al., a randomized, double-blind, placebo-controlled design was used to evaluate the effect of a novel quercetin phytosome formulation in individuals with chronic AR [53]. Sixty-six participants were randomized to receive either standard management with antihistamines and nasal corticosteroids or the same regimen plus a supplement containing 200 mg of quercetin phytosome (Quercefit^®^, complexed with sunflower lecithin for improved bioavailability) for four weeks. Compared with baseline and with the control group, the supplemented group exhibited a significantly greater reduction in nasal congestion, sneezing, and rhinorrhea. Quality of life scores also improved, as measured by RQLQ. Importantly, no adverse events were reported, highlighting the safety of this phytosomal formulation.

This study also assessed biomarkers of inflammation. Serum levels of IL-6, TNF-α, and CRP were significantly decreased in the intervention group after four weeks. These biochemical improvements were consistent with the known anti-inflammatory and mast cell-stabilizing properties of quercetin. The authors concluded that quercetin phytosome may serve as a safe and effective adjunct in the management of AR, particularly when standard therapy provides incomplete relief [53].

In a second trial by the same research group, the effects of the same quercetin phytosome supplement were tested in a cohort of subjects with pollen-induced AR during peak seasonal exposure [161]. This open-label, randomized, controlled study involved 57 participants who were assigned to receive either standard therapy alone or in combination with 200 mg/day of quercetin phytosome for one month. Results echoed the findings of the prior trial: significant reductions in sneezing, nasal itching, and watery rhinorrhea were observed, and overall symptom scores declined more sharply in the quercetin group. The authors emphasized that the formulation’s improved bioavailability—reportedly up to 20 times higher than native quercetin—was likely critical to its clinical effectiveness.

Interestingly, this trial also included a follow-up period of one month after discontinuation. The group that received quercetin supplementation maintained symptom control better than the control group, suggesting a possible sustained or carry-over effect of the intervention. No significant adverse events or biochemical abnormalities were reported, reinforcing the supplement’s safety profile even during high-exposure periods.

Despite these encouraging findings, limitations must be acknowledged. Both studies were relatively small in size and limited to short-term interventions. Moreover, both were conducted by the same research team.

Collectively, these in vivo studies establish a solid foundation for the use of quercetin in allergic disorders, not only through its mast cell-stabilizing properties, but also via its antiangiogenic, immunomodulatory, bronchodilatory, and antioxidant actions.

Quercetin exhibits consistent preclinical efficacy in attenuating mast-cell activation, IgE-mediated responses, and Th2 cytokine signaling. Clinical evidence, while promising, remains preliminary and methodologically limited. Variability in bioavailability and formulation complicates interpretation. Future research should prioritize standardized dosing, pharmacokinetic assessment, and well-powered randomized trials to verify clinical benefit.

Table 7 summarizes the main clinical and preclinical evidence regarding quercetin and its derivatives.

#### 3.5.3. Bioavailability, Safety, and Dosage Considerations

As emphasized earlier, clinical translation is often limited by oral bioavailability. Quercetin exists as aglycone and glycosides; onion glycosides (quercetin-4′-O-glucoside; quercetin-3,4′-O-diglucoside) exhibit superior absorption vs. aglycone [163]. The enzymatic activity of lactase phloridzin hydrolase at the intestinal brush border facilitates the deglycosylation of quercetin glucosides, allowing for subsequent absorption through active or passive mechanisms [164]. Moreover, additional absorption can occur in the colon following microbial degradation of more complex quercetin conjugates such as rutinoside, although this is generally less efficient [165]. Once absorbed, quercetin undergoes rapid and extensive phase II metabolism, including glucuronidation, sulfation, and methylation, in enterocytes and hepatocytes, forming conjugated metabolites such as quercetin-3-glucuronide and isorhamnetin [166]. These metabolites dominate plasma and urinary profiles, and while their bioactivity is still being elucidated, evidence suggests they retain or possibly even surpass the antioxidant potential of the parent compound [167].

Nonetheless, bioavailability remains a significant obstacle. Several studies emphasize the high interindividual variability in quercetin absorption and metabolism. Factors such as gut microbiota composition, genetic polymorphisms in metabolizing enzymes (e.g., uridine 5′-diphospho-glucuronosyltransferases (UGTs), sulfotransferases (SULTs), catechol-O-methyltransferase (COMT)), and dietary context (e.g., co-ingestion with fat, alcohol, or prebiotics) all contribute to discrepancies in circulating levels of quercetin metabolites [164]. For instance, individuals classified as “high absorbers” for quercetin conjugates in the small intestine may exhibit low microbial-derived metabolite production in the colon, and vice versa [164]. This variation underscores the need for personalized approaches when designing supplementation protocols and interpreting clinical outcomes.

In response to the challenge of limited and inconsistent bioavailability, several formulation strategies have been developed. Nanotechnology-based delivery systems—including liposomes, solid lipid nanoparticles, cyclodextrins, and polymeric nanocarriers—have shown promising results in preclinical models. These platforms enhance quercetin solubility, protect it from enzymatic degradation, and promote tissue-specific delivery, thus increasing systemic bioavailability and biological efficacy [168]. For example, nanoencapsulation has been reported to significantly raise quercetin concentrations in plasma and target organs, enabling a more predictable pharmacokinetic profile and sustained release [168]. Similarly, phytosomal and glycosylated formulations are being explored as alternatives to improve stability and gastrointestinal uptake [169].

Regarding safety, quercetin has demonstrated a favorable profile in human clinical trials, with doses up to 1000 mg/day generally well tolerated in short- and medium-term studies [166]. Most reported adverse effects are mild and transient, such as gastrointestinal discomfort or headache, and no major toxicity has been associated with standard supplemental intakes [169]. Importantly, quercetin has received GRAS (Generally Recognized as Safe) status by the FDA for use in food products [164]. However, data on chronic intake beyond 12 weeks remains scarce. Some animal studies have raised concerns about nephrotoxicity under conditions of pre-existing kidney damage and potential estrogenic effects that could promote hormone-dependent tumor growth, especially at high doses [166]. Although these findings have not been replicated in humans, they warrant caution for individuals with compromised renal function or hormone-sensitive pathologies.

Additionally, quercetin’s potential to interfere with drug metabolism via cytochrome P450 inhibition, particularly CYP3A4, poses a risk for pharmacokinetic interactions [170,171]. This could lead to altered efficacy or toxicity of co-administered medications, highlighting the importance of clinical evaluation before recommending high-dose supplementation in patients on polypharmacy.

As for dosage considerations, the optimal therapeutic dose of quercetin remains undetermined. While human studies frequently use doses ranging from 150 mg to 1000 mg/day of the aglycone form, efficacy and safety vary depending on the formulation and population studied [166]. Dabeek and Marra suggest that 500 mg/day may be effective in reducing blood pressure and inflammation but also note that plant-based sources providing lower doses may achieve comparable effects due to higher bioavailability of glycosylated forms [163]. In contrast, high-end dietary intakes in Western populations typically remain between 3–40 mg/day, with 250 mg/day estimated for individuals with high fruit and vegetable consumption [166]. Therefore, supplementation must bridge the gap between dietary intake and pharmacological thresholds, balancing efficacy with safety.

In conclusion, quercetin’s clinical potential hinges on overcoming the challenges posed by its poor and variable bioavailability, establishing long-term safety, and optimizing dosing regimens. While current data support its short-term use as a safe supplement, further research is needed to define standardized, bioavailable formulations and evaluate chronic intake in diverse populations. Advances in delivery technologies and a better understanding of interindividual variability may pave the way for more effective and personalized applications of quercetin in human health.

### 3.6. Epigallocatechin Gallate

#### 3.6.1. Chemical Structure and Dietary Sources of Epigallocatechin Gallate

Epigallocatechin-3-gallate (EGCG) is a catechin (a flavan-3-ol) within the flavonoid family, polyphenolic compounds widely distributed in the plant kingdom. It is considered the most researched flavan-3-ol due to its bioactive properties [172], especially in antioxidant, anti-inflammatory, and anti-allergic contexts. Its molecular formula is C_22_H_18_O_11_ and it has a molecular weight of 458.37 g/mol. The chemical structure of EGCG reveals the presence of eight free hydroxyl groups, which confer a high capacity to donate electrons, interact with ROS (Reactive Oxygen Species), and chelate metal ions, thus explaining its potent antioxidant activity and redox modulation capacity in biological systems [173].

According to data obtained from the specialized database Phenol-Explorer, EGCG has a differential distribution in various food matrices, with tea infusions being the most concentrated sources [172]. In particular, green tea has the highest content with an average value of 27.16 mg/100 mL, followed by Oolong tea with 17.89 mg/100 mL, and black tea with 8.69 mg/100 mL, due to partial or total oxidation processes that affect the stability of catechins. In commercial tea-based infusions, there is a significant reduction in the concentration of EGCG compared to traditional preparations made by directly infusing the leaves. According to data from the Phenol-Explorer database, the average EGCG values in commercial green tea, Oolong tea, and black tea infusions are 8.69 mg/100 mL, 5.11 mg/100 mL, and 1.83 mg/100 mL, respectively. This decrease could be attributed to factors such as dilution, industrial thermal processes, catechin oxidation, and possible degradation during storage and distribution of the final product. In addition, lower concentrations of EGCG have been identified in cocoa-based foods, as well as in various seeds and vegetable fruits, although these represent secondary sources in terms of quantitative dietary intake [174,175].

#### 3.6.2. Preclinical and Clinical Evidence

##### Preclinical Evidence

Preclinical evidence on EGCG in allergies is consistent and robust. In murine OVA-asthma, oral administration of EGCG significantly reduced bronchoalveolar eosinophilia, serum IgE levels, and the expression of Th2 cytokines such as IL-4, IL-5, and IL-13. These effects were accompanied by decreased airway hyperresponsiveness and attenuation of bronchial remodelling, highlighting its potential as an anti-asthmatic agent [54,55].

In human mast-cell cultures, EGCG inhibited degranulation and the release of histamine and TNF-α, confirming its role as a mast-cell stabiliser [56]. Furthermore, lipidomic studies have demonstrated that EGCG reconfigures the mast-cell lipid profile, interfering with second messengers such as diacylglycerols and phospholipids that are essential for exocytosis [57].

In models of AD, both topical application and oral supplementation with EGCG reduced eosinophil and mast-cell infiltration in the skin, decreased IL-4 and thymic stromal lymphopoietin (TSLP) production, and improved skin-barrier integrity [24,176,177].

Similarly, in AR, animal studies have shown that EGCG reduces IgE production, mast-cell infiltration in nasal mucosa, and Th2 cytokine expression, leading to symptomatic improvements such as reduced sneezing and rhinorrhoea. Recent reviews also highlight that EGCG modulates mast-cell activation and downregulates pro-inflammatory cytokine release through inhibition of key signalling pathways such as ERK and NF-κB [58,59,178,179,180,181].

Evidence in urticaria and food-allergy models is more limited; however, in vitro studies indicate that EGCG can inhibit allergen-induced mast-cell degranulation and histamine release, suggesting a preventive role in these reactions as well [57].

Collectively, these preclinical findings establish EGCG as a multi-target modulator of allergic responses, acting at tissue, cellular, and molecular levels.

##### Clinical Evidence

Despite robust preclinical findings, clinical data on EGCG in allergic diseases remain scarce. Some pilot studies with green tea extracts have reported a reduction in symptoms of seasonal AR and improvements in inflammatory parameters, although these trials were limited by small sample sizes and heterogeneity in dosing protocols [16]. In AD, preliminary clinical data suggest that topical EGCG formulations may reduce lesion severity and pruritus, but large-scale randomized controlled trials are still lacking [176].

Larger human studies have primarily addressed safety and pharmacokinetics. Moderate doses of EGCG (100–600 mg/day) have consistently been confirmed as well tolerated, whereas higher doses (≥800 mg/day on an empty stomach) have been associated with hepatotoxicity, leading to regulatory warnings and recommendations for caution [60,182].

Recent systematic reviews emphasize the translational gap between preclinical and clinical research. Although the pharmacological potential of EGCG is well supported by animal and in vitro studies, randomized controlled trials specifically evaluating its efficacy in asthma, AR, or AD are still missing [16,17,183,184,185].

In summary, preclinical data clearly support the preventive and therapeutic potential of EGCG in allergic diseases, including asthma, rhinitis, dermatitis, urticaria and food allergies. However, clinical evidence remains insufficient and requires high-quality trials to establish efficacy, optimal dosing, and long-term safety.

EGCG demonstrates potent antioxidant and immunomodulatory actions in vitro and in vivo, including inhibition of FcεRI-mediated signaling and enhancement of epithelial defense (FcεRI, High-Affinity IgE Receptor). However, human evidence is minimal and largely indirect. Limited bioavailability and instability further constrain its translational potential. Optimized delivery systems and controlled human trials are needed to establish its role in allergic disease prevention or management.

Table 8 summarizes the main clinical and preclinical findings regarding epigallocatechin gallate (EGCG), the principal catechin in green tea.

#### 3.6.3. Bioavailability, Safety, and Dosage Considerations

The oral bioavailability of EGCG is limited due to its chemical instability, first-pass metabolism, and rapid conjugation in the liver and intestine. After ingesting green tea or purified extracts, only 0.1–1.0% of the ingested dose reaches the systemic circulation in free form [188]. Pharmacokinetic studies show that peak plasma concentration is reached within 1–2 h after ingestion, with elimination in less than 24 h. Co-administration with fatty foods, vitamin C, or omega-3 oils may improve its stability and absorption, while fasting intake increases exposure but also the risk of adverse effects [10].

To overcome these limitations, formulation strategies such as nanoemulsions, liposomes, phytosomes, and polymeric nanoparticles have been developed, which increase the stability and bioavailability of EGCG, achieving higher plasma concentrations and more consistent biological effects [187].

In terms of safety, the EFSA concluded that doses of ≥800 mg/day of EGCG on an empty stomach are associated with significant elevations in liver transaminases, indicating a risk of hepatotoxicity [182]. In contrast, moderate doses of 100–600 mg/day, administered with food, are considered safe in most clinical trials [186]. The most common adverse effects include mild gastrointestinal discomfort, dizziness, or anemia in isolated cases.

In practical terms, the consumption of green tea as a beverage provides approximately 50–100 mg of EGCG per cup, which represents a safe and common intake among Asian populations. For therapeutic purposes, purified and standardized extracts such as Teavigo^®^ (containing ≥94% EGCG) have been used in clinical studies at daily doses ranging from 200 to 400 mg. Importantly, administration together with food is always recommended, since intake in the fasting state at higher doses (≥800 mg/day) has been associated with hepatotoxicity, whereas moderate doses with meals are considered safe and well tolerated [186].

In summary, EGCG has low bioavailability, which limits its clinical efficacy, but new and improved formulations offer promising prospects. Safety is well established at moderate doses, while high doses on an empty stomach should be avoided. Clinical implementation requires balancing efficacy, bioavailability, and safety, with liver monitoring in prolonged treatments.

### 3.7. Mechanisms of Action of Dietary Bioactive Compounds in Allergy Prevention

#### 3.7.1. General Immunological Mechanisms

Despite their chemical diversity, ranging from polyunsaturated fatty acids and vitamins to flavonoids and polyphenolic alkaloids, dietary bioactive compounds converge on overlapping immunological targets that are central to the pathophysiology of allergic diseases. Allergic disorders are driven by an imbalance toward Th2-dominated immunity, characterized by excessive production of cytokines such as IL-4, IL-5, and IL-13, promotion of IgE class switching, eosinophil recruitment, and mast-cell activation. Many bioactives act precisely at this interface, shifting the immune balance away from Th2 polarization while promoting the activity of regulatory Tregs and the secretion of IL-10, a cytokine that supports tolerance to otherwise harmless antigens [28,32,35,36,37,189,190,191,192,193,194,195,196,197,198,199].

At the intracellular signaling level, several compounds—including curcumin, quercetin, ginger bioactives, and EGCG—consistently inhibit the activation of NF-κB and MAPK pathways, master regulators of pro-inflammatory transcriptional programs. By blocking these hubs, bioactives reduce downstream cascades that drive eosinophilic inflammation, airway hyperresponsiveness, and chronic tissue remodeling [35,36,37,139,189,190,191,200]. These actions parallel those of omega-3 fatty acids, which act upstream at the lipid mediator level by replacing arachidonic acid in membranes and promoting the synthesis of SPMs [25,191]. Vitamin D, although structurally distinct, converges on similar outcomes through Vitamin D receptor (VDR)-mediated transcriptional regulation, leading to tolerogenic dendritic cells and suppression of Th2/Th17 activity [28,32,193].

A second major hub is mast-cell stabilization, crucial because mast cells orchestrate the early-phase allergic response via release of histamine, tryptase, prostaglandins, and leukotrienes. Quercetin and EGCG are the most potent mast-cell stabilizers, directly inhibiting FcεRI-dependent degranulation and signaling pathways such as Lyn/PLCγ/ERK [156]. Curcumin, gingerols, and certain omega-3 derivatives also modulate mast-cell activity, either indirectly via cytokine modulation or directly through PPAR-γ and cAMP pathways [139,190,191,201]. This broad suppression of mast-cell activation underpins the consistent clinical observation of reduced pruritus, rhinorrhea, and wheezing in intervention studies.

Another shared mechanism is the reinforcement of epithelial barriers. The integrity of skin, airway, and intestinal epithelia is critical in preventing allergen translocation and microbial adjuvants that exacerbate immune activation. Vitamin D and curcumin are particularly effective in upregulating tight-junction proteins and antimicrobial peptides (e.g., cathelicidin), whereas ginger bioactives and EGCG also contribute to epithelial protection through indirect antioxidant and anti-inflammatory effects [93,94,197,202,203,204]. This barrier-enhancing activity is clinically relevant in AD and food allergy, where increased permeability facilitates sensitization.

Finally, oxidative stress represents a unifying pathological feature across allergic conditions, amplifying NF-κB activation, disrupting barriers, and perpetuating inflammation. Curcumin, ginger bioactives, and EGCG are particularly potent antioxidants, activating the Nrf2/HO-1 (Nuclear Factor Erythroid 2–Related Factor 2/Heme Oxygenase-1) pathway and boosting endogenous defenses such as superoxide dismutase, glutathione peroxidase, and catalase [200,203,204,205]. Omega-3 fatty acids indirectly reduce oxidative stress by lowering reactive oxygen species generated via eicosanoid metabolism, while vitamin D influences redox balance through regulation of mitochondrial function and antimicrobial defense [28,202].

Taken together, these common mechanisms—Th2 suppression, Treg induction, NF-κB/MAPK inhibition, mast-cell stabilization, epithelial barrier reinforcement, and oxidative stress reduction—create a converging framework through which structurally diverse dietary bioactives can attenuate allergic inflammation. This convergence also highlights the potential for additive or synergistic effects when multiple bioactives are consumed as part of a varied diet.

Taken together, these findings highlight both compound-specific and overlapping mechanisms, with recurrent modulation of NF-κB, MAPK, and Treg pathways.

#### 3.7.2. Omega-3 Polyunsaturated Fatty Acids (PUFAs)

Omega-3 PUFAs exert their preventive effects in allergic diseases mainly through their role as precursors of SPMs, such as resolvins and protectins, which actively terminate inflammation and restore tissue homeostasis. These mediators are impaired in severe asthma and food allergy, and supplementation with EPA and DHA enhances their biosynthesis in both preclinical and clinical studies [189,191]. Another distinctive aspect is the influence of the dietary omega-6:omega-3 ratio: higher ratios are consistently linked to increased allergic risk, while higher genetically determined levels of DHA appear protective [25,192]. Maternal supplementation during pregnancy and lactation has been associated with reduced risk of IgE-mediated sensitization and AD in children, although the effect often diminishes later in childhood [25,26,192]. Overall, the evidence suggests that omega-3 intake during critical developmental windows may offer preventive benefits, though the magnitude and persistence remain uncertain.

#### 3.7.3. Vitamin D

The unique role of vitamin D in allergic disease prevention lies in its ability to act through the VDR, regulating transcription of genes involved in immune tolerance and barrier integrity. Preclinical studies highlight its modulation of pathways such as STAT3/AKT/mTOR, particularly in AD models, leading to improved barrier function and reduced disease severity [32,90]. During gestation and infancy, adequate vitamin D levels contribute to fetal immune programming, lowering the risk of subsequent sensitization [33,34,88,89,99]. However, clinical evidence remains heterogeneous: supplementation reduces AD severity in deficient patients [96,97,98,195], yet randomized controlled trials have not consistently demonstrated a reduction in asthma or food allergy incidence in unselected populations [29,93,94,95,100]. Genetic polymorphisms in vitamin D–binding proteins further modulate its bioavailability and may explain conflicting results [102].

#### 3.7.4. Curcumin

Curcumin contributes to allergy prevention mainly through actions on epithelial integrity and mast-cell regulation. It downregulates IL-4, IL-5, IL-13, and TNF-α while enhancing IL-10, thus favoring a tolerogenic environment. At the cellular level, curcumin inhibits histamine release and mast-cell degranulation, while improving barrier function in epithelial models. These effects are mediated by the modulation of NF-κB and MAPK signaling pathways, placing curcumin as a relevant modulator of both inflammatory and structural components of allergic disease [35,36,37,196,197,198,199].

#### 3.7.5. Ginger

The main bioactives in ginger, [6]-gingerol and [6]-shogaol, act through multiple signaling cascades beyond the common hubs of Th2 and NF-κB. They inhibit phosphodiesterase, modulate PI3K/Akt and JAK/STAT/FOXO pathways, and activate AMPK (AMP-Activated Protein Kinase), contributing to airway smooth muscle relaxation and immune balance. Ginger also enhances antioxidant defenses via Nrf2/HO-1 and regulates epithelial homeostasis through Wnt/β-catenin signaling. Collectively, these mechanisms underline ginger’s distinctive ability to integrate metabolic and immunological regulation in allergy prevention [52,139,150,200,203,204,205,206,207,208,209,210].

#### 3.7.6. Quercetin

Quercetin is characterized by strong mast-cell stabilization, reducing histamine, tryptase, β-hexosaminidase, IL-4, and TNF-α release. These effects are consistent with the signaling mechanisms summarized in Table 1, notably Lyn-kinase inhibition and NF-κB modulation.. In addition, quercetin modulates pseudoallergic responses mediated byMas-related G protein-coupled receptor member X2, and contributes to balancing Th1/Th2 responses and promoting Treg activity, ultimately lowering IgE levels [45,156,157,158,159,160,162,211].

#### 3.7.7. Epigallocatechin Gallate (EGCG)

EGCG exerts its antiallergic effects primarily by targeting FcεRI-mediated mast-cell activation. EGCG modulates FcεRI-mediated mast-cell signaling pathways consistent with those outlined in Table 1, while adding potent antioxidant effects via Nrf2/HO-1 activation. In parallel, EGCG neutralizes reactive oxygen species and activates Nrf2/HO-1, boosting endogenous antioxidant defenses. It also reprograms mast-cell lipid signaling and decreases IgE–allergen binding through polyphenol–protein complexes, providing an additional safeguard at the sensitization phase [24,54,56,57,178,212,213].

## 4. Conclusions and Future Research Lines

The evidence reviewed highlights the potential of bioactive dietary compounds such as omega-3 fatty acids, vitamin D, curcumin, ginger bioactives, quercetin and epigallocatechin gallate in modulating immune responses and contributing to the prevention or attenuation of allergic diseases. Although their chemical structures and dietary sources differ, these compounds converge on common mechanisms including mast-cell stabilization, suppression of Th2 polarization and IgE-mediated inflammation, enhancement of regulatory T-cell activity and reinforcement of epithelial and antioxidant defenses.

Despite consistent findings in preclinical models, translation into clinical practice remains limited. Human trials are often small, heterogeneous in design and duration, and use variable formulations and dosages. Low and inconsistent bioavailability, especially for polyphenols, and the influence of baseline status and interindividual variability, particularly in the case of vitamin D, further complicate the establishment of general recommendations. The heterogeneity of study populations, differences in methodologies, and potential publication bias toward positive results also limit the reliability and comparability of current evidence. Moreover, translating preclinical mechanisms into clinically meaningful outcomes remains challenging, as experimental models frequently fail to capture the complexity of human immune responses.

Future studies should aim to overcome these limitations by addressing the lack of standardization in formulations and doses, which hinders comparisons across trials, and by conducting long-term randomized controlled trials with sufficient statistical power to evaluate meaningful clinical endpoints such as disease onset or sustained remission. Particular attention should be given to interventions during pregnancy and early life, as these represent critical windows for immune programming. Furthermore, the exploration of combined or synergistic approaches is warranted, since several of these bioactives share overlapping mechanisms that could enhance efficacy while reducing the required dose. Precision nutrition strategies also represent an important avenue, as genetic variability, baseline nutritional status, and host-related factors strongly influence responsiveness to supplementation. Finally, although these compounds are generally regarded as safe, systematic evaluation of long-term safety and tolerability is necessary, particularly at high doses or when using formulations designed to overcome bioavailability limitations.

In conclusion, dietary bioactive compounds hold promise as accessible and sustainable tools for allergy prevention and management. Nonetheless, translation from mechanistic and preclinical evidence into clinical practice requires rigorous, large-scale, and well-standardized trials to establish efficacy, safety, and optimal use across different populations.

## Figures and Tables

**Table 1:** Overview of dietary bioactive compounds with potential roles in allergy prevention.

Compound	Chemical Class/Main Structure	Dietary Sources	Typical Intake or Supplementation Range	Bioavailability	Main Molecular Targets and Pathways	Key Immunological Effects	References
**Omega-3 fatty acids (ALA, EPA, DHA, DPA, SDA)**	Polyunsaturated fatty acids (C18–C22, cis double bonds)	Oily fish, fish/krill oil, microalgae, flaxseed, chia, walnuts	250–500 mg/day EPA + DHA for general health; ≥1200 mg/day in prevention trials	Moderate; higher for triglyceride/phospholipid forms than ethyl esters; improved with emulsions or nanoformulations	Inhibition of NF-κB and MAPK pathways, activation of PPAR-γ, generation of SPMs	Reduction in Th2 cytokines (IL-4, IL-5, IL-13), increase in regulatory T cells, production of specialized pro-resolving mediators, lower IgE	[25,26,27]
Vitamin D (D_2_, D_3_)	Fat-soluble secosteroids	Sun exposure, oily fish, eggs, fortified foods, UV-treated mushrooms	1000–2000 IU/day commonly recommended; up to 5000 IU/day tested	Variable; influenced by fat intake, obesity, genetics; improved with micellar or nanoemulsion forms	Activation of VDR signaling, induction of tolerogenic dendritic cells, inhibition of NF-κB and MAPK pathways	Promotes tolerogenic dendritic cells, increases IL-10, enhances Treg activity, reduces IgE synthesis, improves epithelial barrier	[28,29,30,31,32,33,34]
Curcumin	Polyphenolic curcuminoid (diferuloylmethane)	Turmeric rhizome, curry powders, standardized extracts	500–2000 mg/day in supplements	Very low orally; improved with piperine, phospholipid complexes, nanoparticles	Inhibition of NF-κB and MAPK pathways, activation of Nrf2/HO-1 signaling, stabilization of mast cells	Inhibition of NF-κB and MAPK, reduction in Th2 cytokines, stabilization of mast cells, improvement of barrier integrity	[35,36,37,38,39,40,41,42]
Ginger bioactives (6-gingerol, 6-shogaol)	Phenolic alkanones	Fresh or dried ginger, powdered rhizome, teas, extracts	500 mg–2 g/day standardized extract (≥5% gingerols)	Moderate; metabolism to gingerdiols and conjugates; improved with liposomal or self-emulsifying systems	Inhibition of NF-κB signaling, modulation of cAMP pathways, activation of Nrf2/HO-1, regulation of HDAC2 and HDAC3	Reduction in Th2 and Th1 cytokines, inhibition of mast-cell degranulation, activation of Nrf2/HO-1, increase in IL-10	[43,44]
Quercetin	Flavonol (3,3′,4′,5,7-pentahydroxyflavone)	Onions, apples, kale, berries, tea, wine	100–1000 mg/day in trials	Low; glycosylated forms more bioavailable; improved in phytosome formulations	Inhibition of NF-κB, blockade of Lyn/PLCγ/ERK1/2 signaling, downregulation of PI3K-AKT pathway, stabilization of mast cells	Mast-cell stabilization, inhibition of histamine and TNF-α release, blockade of Lyn/PLCγ/ERK1/2 pathways, rebalancing of Th1/Th2 responses, enhancement of Treg activity	[45,46,47,48,49,50,51,52,53]
Epigallocatechin gallate (EGCG)	Flavan-3-ol catechin	Green tea, supplements	150–800 mg/day (as tea catechins or capsules)	Limited due to metabolism; improved with encapsulation or phospholipid complexes	Inhibition of NF-κB and MAPK pathways, suppression of FcεRI signaling, modulation of lipid mediators	Mast-cell stabilization, inhibition of FcεRI signaling, attenuation of airway inflammation, reduction in oxidative stress	[54,55,56,57,58,59,60]

ALA, α-linolenic acid; cAMP, cyclic adenosine monophosphate; DHA, docosahexaenoic acid; DPA, docosapentaenoic acid; EGCG, epigallocatechin gallate; EPA, eicosapentaenoic acid; FcεRI, High-Affinity IgE Receptor; IgE, immunoglobulin E; IL, interleukin; Lyn/PLCγ/ERK1/2, Lyn tyrosine kinase/phospholipase C gamma/extracellular signal-regulated kinase 1 and 2; MAPK, mitogen-activated protein kinase; NF-κB, nuclear factor kappa B; Nrf2/HO-1, Nuclear Factor Erythroid 2 Related Factor 2/Heme Oxygenase 1; PPAR-γ, peroxisome proliferator–activated receptor gamma; SDA, stearidonic acid; TNF-α, Tumor Necrosis Factor Alpha; SPMs, specialized pro-resolving mediators; Th1/Th2, T helper Cells Type 1/Type 2; Treg, regulatory T cells; VDR, vitamin D receptor.

**Table 2:** Mayor Omega-3 and their structures [62].

Fatty Acid	Molecular Formula	Carbon Atoms and Double Bonds	Key Characteristics
ALA	C18:3*n*-3	18 carbons, 3 double bonds (cis)	Essential; the human body cannot synthesize it.
EPA	C20:5*n*-3	20 carbons, 5 double bonds	Well-known for its anti-inflammatory properties.
DHA	C22:6*n*-3	22 carbons, 6 double bonds	Longest-chain fatty acid, crucial for the nervous system and retina.
SDA	C18:4*n*-3	18 carbons, 4 double bonds	Intermediate in EPA biosynthesis; an alternative to marine sources.
DPA	C22:5*n*-3	22 carbons, 5 double bonds	Present in mammalian tissues; has unique functions.

ALA, α-linolenic acid; EPA, eicosapentaenoic acid; DHA, docosahexaenoic acid; SDA, stearidonic acid; DPA, docosapentaenoic acid. This table was independently developed by the authors using information synthesized from multiple sources, including Shahidi & Ambigaipalan (2018, Annu. Rev. Food Sci. Technol. 9:345–381 [62]), but it does not reproduce or directly adapt any existing table.

**Table 3:** Immunomodulatory mechanisms and clinical relevance of omega-3 fatty acids.

Study Type	Model/Population	Target Condition	Main Outcomes	Effect Direction	Quality	References
Clinical (RCTs, meta-analyses, epidemiological studies)	Pregnant/lactating women, infants, children	Eczema, food allergy, asthma, allergic rhinitis	↓ Risk of eczema, food sensitization, and IgE-mediated allergies (not consistent across all studies); modest reduction in allergic sensitization; stronger effects with high-dose or long-duration supplementation (≥1200 mg/day).	Positive/Mixed	Moderate	[27,69,70,71]
Preclinical (in vivo/in vitro)	Animal models (mice, rats), in vitro immune cell assays (dendritic, T, B, mast cells)	Food allergy, asthma, airway inflammation	↓ IgE, IL-4, IL-5, TNF-α, prostaglandins, leukotrienes; ↑ SPMs (resolvins, protectins); Th2 modulation; reduced airway eosinophilia and allergic symptoms. Mechanistic evidence of immune regulation and anti-inflammatory activity.	Positive	High	[25,26,68]

IgE, immunoglobulin E; IL, interleukin; TNF-α, tumor necrosis factor alpha; SPMs, specialized pro-resolving mediators; RCTs, randomized controlled trials.

**Table 4:** Immunomodulatory mechanisms and clinical relevance of vitamin D in allergic and inflammatory diseases.

Study Type	Model/Population	Target Condition	Main Outcomes	Effect Direction	Quality	References
Clinical	Children, adults, pregnant women, infants (RCTs, meta-analyses, cohort studies)	Asthma, rhinitis, recurrent wheezing, atopic dermatitis, food allergy	Mild benefit in asthma and rhinitis only in severe deficiency (<10 ng/mL); ↑ IL-10, no effect on IgE/eosinophils/FeNO. In atopic dermatitis, ↓ SCORAD/EASI (−0.41 to −0.50), ↑ VDR and LL-37, improved barrier integrity. No consistent reduction in food allergy risk; possible modulation by VDBP polymorphisms.	Neutral to positive	Low–Moderate to Moderate–High	[28,30,31,32,33,34,92,97,99,100,101,102,103,104,105,106,107,108,109,110,111]
Preclinical (in vitro)	Dendritic, T and B lymphocytes, epithelial and keratinocyte cell lines	Immunomodulation and epithelial defense	Activation of VDR → tolerogenic dendritic phenotype; ↑ IL-10 and Treg; ↓ Th2/Th17 cytokines (IL-4, IL-13, IL-22); ↓ IgE synthesis; ↑ antimicrobial peptides and barrier proteins (tight junctions, cathelicidin).	Positive	High	[28,91,92]
Preclinical (in vivo—allergic models)	Murine models of OVA-induced asthma, DNCB/OVA-induced dermatitis, and dietary antigen–sensitized food allergy (BALB/c, NC/Nga mice)	Airway, skin, and intestinal allergic inflammation; epithelial barrier dysfunction	Vitamin D or calcifediol supplementation attenuated allergic inflammation (↓ eosinophilia, IL-4, IL-5, IL-13, IL-33); enhanced tolerance (↑ Treg, IL-10); inhibited NF-κB and STAT3/AKT/mTOR; restored epithelial integrity (normalized VDR and VDBP, ↓ aquaporin-3, improved barrier function).	Positive	High	[93,98]

25(OH)D, 25-hydroxyvitamin D; AKT, protein kinase B; DNCB, 2,4-dinitrochlorobenzene; EASI, Eczema Area and Severity Index; FeNO, fractional exhaled nitric oxide; IgE, immunoglobulin E; IL, interleukin; mTOR, mechanistic target of rapamycin; NF-κB, nuclear factor kappa B; OVA, ovalbumin; RCTs, randomized controlled trials; SCORAD, Scoring Atopic Dermatitis index; STAT3, signal transducer and activator of transcription 3; Th2/Th17, T helper cells type 2/type 17; Treg, regulatory T cells; VDBP, vitamin D–binding protein; VDR, vitamin D receptor; LL-37, human cathelicidin antimicrobial peptide.

**Table 5:** Immunomodulatory mechanisms and clinical relevance of curcumin in allergic and inflammatory diseases.

Study Type	Model/Population	Target Condition	Main Outcomes	Effect Direction	Quality	References
Clinical	Adults with perennial allergic rhinitis; patients with mild AR; AD patients (topical/oral); chronic urticaria (pilot); moderate–severe asthma (pilot)	Allergic rhinitis, atopic dermatitis, chronic spontaneous urticaria, asthma	AR: Reduce TNSS, sneezing, rhinorrhea; Reduce IL-4, IL-8, TNF-α; increase IL-10, sICAM; improved nasal airflow. AD/eczema: Reduce erythema, scaling, pruritus; good tolerability. Urticaria: Reduce wheals and pruritus (pilot). Asthma: pilot RCT underway/reported with symptom and biomarker endpoints	Positive	Moderate Consistent symptom improvements in AR and AD; evidence in urticaria/asthma is preliminary and heterogeneous	[35,37,39,121,122]
Preclinical (in vivo)	Mice (OVA-induced asthma); murine AD models	Asthma, atopic dermatitis	Reduce IgE and Th2 cytokines (IL-4, IL-13); inhibition of NF-κB/STAT6/MAPK; reduced airway eosinophilia and skin inflammation; improved barrier-related outcomes	Positive	High reproducible anti-allergic and anti-inflammatory effects across murine models	[38,40]
Preclinical (in vitro)	Mast cells, keratinocytes, airway epithelial cells	Mast-cell activation, cytokine release, epithelial barrier	Reduce Histamine/β-hexosaminidase release; Reduce IL-6/IL-8; inhibition of NF-κB/AP-1; antioxidant and barrier-supportive effects	Positive	Moderate Strong mechanistic support; translational impact depends on formulation/bioavailability	[42,120]

AD, atopic dermatitis; AP-1, activator protein 1; AR, allergic rhinitis; IgE, immunoglobulin E; IL, interleukin; MAPK, mitogen-activated protein kinase; NF-κB, nuclear factor kappa B; OVA, ovalbumin; RCT, randomized controlled trial; sICAM, soluble intercellular adhesion molecule; STAT6, signal transducer and activator of transcription 6; Th2, T helper type 2; TNF-α, tumor necrosis factor alpha; TNSS, Total Nasal Symptom Score.

**Table 6:** Summary of clinical and experimental evidence on the immunomodulatory effects of ginger bioactives.

Study Type	Model/Population	Target Condition	Main Outcomes	Effect Direction	Quality (Justification)	References
Clinical	Patients with atopic dermatitis (n = 44), allergic rhinitis (n = 80), asthma (n = 32), and pharmacokinetic studies in asthma patients	Atopic dermatitis, allergic rhinitis, asthma	↓ NF-κB and cytokine release; ↓ pruritus (55%) and improved skin barrier; ↓ TNSS and RQLQ, ↑ nasal airflow; ↑ ACT score and QoL; defined pharmacokinetics of gingerols/shogaols in humans.	Positive or neutral–supportive	High (randomized controlled trials, human PK studies, consistent evidence)	[143,146,147,148]
Preclinical	Caco-2 intestinal epithelial cells; OVA- and HDM-induced murine asthma models; diabetic and neuropathic rat models	Asthma, allergic inflammation, gut–brain/immune axis	↓ HDAC2/3 and NF-κB; ↑ ALDH1A1, RA signaling, and Treg activation; reduced Th2 cytokines, eosinophilia, and mucus; enhanced antioxidant defenses; modulation of gut microbiota, mitochondrial metabolism, and gut–lung axis.	Positive	High to moderate (in vivo and in vitro, mechanistic data replicated across models)	[43,139,140,141,142,149]

ACT, Asthma Control Test; ALDH1A1, aldehyde dehydrogenase 1 family member A1; HDAC2/3, histone deacetylase 2/3; HDM, house dust mite; IL, interleukin; NF-κB, nuclear factor kappa B; OVA, ovalbumin; PK, pharmacokinetics; QoL, quality of life; RA, retinoic acid; RQLQ, Rhinoconjunctivitis Quality of Life Questionnaire; Th2, T helper type 2; TNSS, Total Nasal Symptom Score; Treg, regulatory T cells.

**Table 7:** Immunomodulatory mechanisms and clinical relevance of quercetin in allergic and inflammatory diseases.

Study Type	Model/Population	Target Condition	Main Outcomes	Effect Direction	Quality	References
Clinical	Adults with allergic rhinitis and mild allergic reactions (RCTs and pilot trials)	Allergic rhinitis, allergic reactions, early allergy prevention	↓ Nasal and ocular symptom scores, ↓ serum histamine, ↓ IL-8 and TNF-α; improved quality of life. Quercetin-containing supplements and bioavailable formulations (e.g., EMIQ) were well tolerated and effective.	Positive	Moderate	[53,161]
Preclinical (in vivo)	Mice and rats (OVA-induced allergic rhinitis, asthma, conjunctivitis models)	Allergic rhinitis, asthma, conjunctivitis	↓ IgE, IL-4, IL-5, TNF-α, IL-17, VEGF, bFGF; inhibition of NF-κB, Lyn kinase, COX-2 and RELA pathways; reduced sneezing, nasal rubbing and airway inflammation. Nanoparticle (chitosan-based) and glycosylated forms (EMIQ) markedly enhanced efficacy.	Positive	High (enhanced forms)/Moderate (standard quercetin)	[46,47,49,51,52,162]
Preclinical (in vitro)	Human and murine mast cells, airway epithelial cells	Mast-cell activation, angiogenesis, cytokine release	↓ Degranulation and Ca^2+^ influx, inhibition of Lyn/PLCγ/IP_3_R signaling; ↓ VEGF, IL-6, IL-8 secretion. Findings elucidate quercetin’s inhibition of mast-cell-driven inflammation and angiogenesis.	Positive	Moderate	[46,49,160]

bFGF, basic fibroblast growth factor; COX-2, cyclooxygenase-2; EMIQ, enzymatically modified isoquercitrin; IgE, immunoglobulin E; IL, interleukin; IP_3_R, inositol-1,4,5-trisphosphate receptor; Lyn, tyrosine-protein kinase Lyn; NF-κB, nuclear factor kappa-B; OVA, ovalbumin; PLCγ, phospholipase C gamma; RCT, randomized controlled trial; RELA, v-rel avian reticuloendotheliosis viral oncogene homolog A (p65 subunit of NF-κB); TNF-α, tumor necrosis factor alpha; VEGF, vascular endothelial growth factor.

**Table 8:** Immunomodulatory mechanisms and clinical relevance of epigallocatechin gallate (EGCG) in allergic and inflammatory diseases.

Study Type	Model/Population	Target Condition	Main Outcomes	Effect Direction	Quality	References
Clinical	Adults with seasonal allergic rhinitis (pilot studies); patients with atopic dermatitis (topical formulations); healthy volunteers (safety and pharmacokinetics)	Allergic rhinitis, atopic dermatitis, safety/bioavailability	Reduction in nasal and ocular symptoms and inflammatory parameters in small trials; topical EGCG reduced pruritus and lesion severity; moderate doses (100–600 mg/day) well tolerated; ≥800 mg/day fasting associated with hepatotoxicity; low oral bioavailability improved by novel formulations (nanoemulsions, liposomes, phytosomes)	Positive but limited	Moderate—preliminary efficacy data; high-quality safety evidence	[60,176,186,187]
Preclinical (in vivo)	Mice (OVA-induced asthma, allergic rhinitis); murine models of atopic dermatitis	Asthma, allergic rhinitis, atopic dermatitis	↓ IgE, ↓ IL-4, IL-5, IL-13; reduced eosinophil and mast-cell infiltration; ↓ airway hyperresponsiveness and bronchial remodeling; ↓ TSLP; improved skin-barrier integrity; reduced sneezing and rhinorrhea; inhibition of NF-κB and ERK pathways	Positive	High—consistent animal studies with robust anti-allergic effects	[59,180,181]
Preclinical (in vitro)	Human mast cells; lipidomic studies of mast-cell activation	Mast-cell activation, mediator release	Inhibition of degranulation; ↓ histamine and TNF-α release; reprogramming of mast-cell lipid profile; ↓ IL-4, IL-5 secretion; modulation of pro-inflammatory signaling	Positive	High—mechanistic evidence supporting biological plausibility	[56,57,58]

EGCG, epigallocatechin gallate; ERK, extracellular signal-regulated kinase; IgE, immunoglobulin E; IL, interleukin; NF-κB, nuclear factor kappa B; OVA, ovalbumin; TSLP, thymic stromal lymphopoietin; TNF-α, tumor necrosis factor alpha.

## Data Availability

No new data were created or analyzed in this study. Data sharing is not applicable to this article.

## Abbreviations

The following abbreviations are used in this manuscript:

ACT	Asthma Control Test
ACD	Allergic Contact Dermatitis
AD	Atopic Dermatitis
AMPK	AMP-Activated Protein Kinase
AR	Allergic Rhinitis
cAMP	Cyclic Adenosine Monophosphate
COX	Cyclooxygenase
CRP	C-Reactive Protein
CQAB	Curcumin and Ashwagandha Bioactive Complex
EASI	Eczema Area and Severity Index
EGCG	Epigallocatechin Gallate
ERK1/2	Extracellular Signal-Regulated Kinases 1/2
FDA	Food and Drug Administration
FcεRI	High-Affinity IgE Receptor
FeNO	Fractional Exhaled Nitric Oxide
FOXO	Forkhead Box O
HDAC	Histone Deacetylase
IL	Interleukin
JAK/STAT	Janus Kinase/Signal Transducer and Activator of Transcription
Lyn	Lyn tyrosine kinase
MAPK	Mitogen-Activated Protein Kinase
NF-κB	Nuclear Factor Kappa B
Nrf2/HO-1	Nuclear Factor Erythroid 2–Related Factor 2/Heme Oxygenase-1
OVA	Ovalbumin
PCA	Passive Cutaneous Anaphylaxis
PI3K/Akt	Phosphoinositide 3-Kinase/Protein Kinase B
PLCγ	Phospholipase C gamma
QCS	Quercetin-Loaded Chitosan Nanoparticles
RA	Retinoic Acid
RQLQ	Rhinoconjunctivitis Quality of Life Questionnaire
ROS	Reactive Oxygen Species
SAD	Stearidonic Acid
SCORAD	Scoring Atopic Dermatitis
Th1/Th2	T Helper Cells Type 1/Type 2
TNF-α	Tumor Necrosis Factor Alpha
TNSS	Total Nasal Symptom Score
Treg	Regulatory T Cell
UVB	Ultraviolet B
VDR	Vitamin D receptor
